# International cohort study indicates no association between alpha-1 blockers and susceptibility to COVID-19 in benign prostatic hyperplasia patients

**DOI:** 10.3389/fphar.2022.945592

**Published:** 2022-09-14

**Authors:** Akihiko Nishimura, Junqing Xie, Kristin Kostka, Talita Duarte-Salles, Sergio Fernández Bertolín, María Aragón, Clair Blacketer, Azza Shoaibi, Scott L. DuVall, Kristine Lynch, Michael E. Matheny, Thomas Falconer, Daniel R. Morales, Mitchell M. Conover, Seng Chan You, Nicole Pratt, James Weaver, Anthony G. Sena, Martijn J. Schuemie, Jenna Reps, Christian Reich, Peter R. Rijnbeek, Patrick B. Ryan, George Hripcsak, Daniel Prieto-Alhambra, Marc A. Suchard

**Affiliations:** ^1^ Department of Biostatistics, Bloomberg School of Public Health, Johns Hopkins University, Baltimore, MD, United States; ^2^ Centre for Statistics in Medicine, Nuffield Department of Orthopaedics, Rheumatology and Musculoskeletal Sciences, Oxford University, Oxford, United Kingdom; ^3^ Real World Solutions, IQVIA, Cambridge, MA, United States; ^4^ The OHDSI Center at The Roux Institute, Northeastern University, Portland, ME, United States; ^5^ Fundació Institut Universitari Per a la Recerca a l'Atenció Primària de Salut Jordi Gol i Gurina (IDIAPJGol), Barcelona, Spain; ^6^ Observational Health Data Analytics, Janssen Research and Development, Titusville, NJ, United States; ^7^ VA Informatics and Computing Infrastructure, VA Salt Lake City Health Care System, Salt Lake City, UT, United States; ^8^ Department of Internal Medicine, University of Utah School of Medicine, Salt Lake City, UT, United States; ^9^ Tennessee Valley Healthcare System, Veterans Affairs Medical Center, Nashville, TN, United States; ^10^ Department of Biomedical Informatics, Vanderbilt University Medical Center, Nashville, TN, United States; ^11^ Department of Biomedical Informatics, Columbia University, New York, NY, United States; ^12^ Division of Population Health and Genomics, University of Dundee, Dundee, United Kingdom; ^13^ Department of Public Health, University of Southern Denmark, Southern Denmark, Denmark; ^14^ Department of Preventive Medicine and Public Health, Yonsei University College of Medicine, Seoul, South Korea; ^15^ Quality Use of Medicines and Pharmacy Research Centre, Clinical and Health Sciences, University of South Australia, Adelaide, Australia; ^16^ Department of Medical Informatics, Erasmus University Medical Center, Rotterdam, Netherlands; ^17^ Department of Biostatistics, UCLA Fielding School of Public Health, University of California, Los Angeles, Los Angeles, CA, United States; ^18^ Department of Computational Medicine, David Geffen School of Medicine at UCLA, University of California, Los Angeles, Los Angeles, CA, United States; ^19^ Department of Human Genetics, David Geffen School of Medicine at UCLA, University of California, Los Angeles, Los Angeles, CA, United States

**Keywords:** treatment for SARS CoV-2, observational study, electronic health records, federated data model, causal inference, open science

## Abstract

**Purpose:** Alpha-1 blockers, often used to treat benign prostatic hyperplasia (BPH), have been hypothesized to prevent COVID-19 complications by minimising cytokine storm release. The proposed treatment based on this hypothesis currently lacks support from reliable real-world evidence, however. We leverage an international network of large-scale healthcare databases to generate comprehensive evidence in a transparent and reproducible manner.

**Methods:** In this international cohort study, we deployed electronic health records from Spain (SIDIAP) and the United States (Department of Veterans Affairs, Columbia University Irving Medical Center, IQVIA OpenClaims, Optum DOD, Optum EHR). We assessed association between alpha-1 blocker use and risks of three COVID-19 outcomes—diagnosis, hospitalization, and hospitalization requiring intensive services—using a prevalent-user active-comparator design. We estimated hazard ratios using state-of-the-art techniques to minimize potential confounding, including large-scale propensity score matching/stratification and negative control calibration. We pooled database-specific estimates through random effects meta-analysis.

**Results:** Our study overall included 2.6 and 0.46 million users of alpha-1 blockers and of alternative BPH medications. We observed no significant difference in their risks for any of the COVID-19 outcomes, with our meta-analytic HR estimates being 1.02 (95% CI: 0.92–1.13) for diagnosis, 1.00 (95% CI: 0.89–1.13) for hospitalization, and 1.15 (95% CI: 0.71–1.88) for hospitalization requiring intensive services.

**Conclusion:** We found no evidence of the hypothesized reduction in risks of the COVID-19 outcomes from the prevalent-use of alpha-1 blockers—further research is needed to identify effective therapies for this novel disease.

## Introduction

As the number of infections with severe acute respiratory syndrome coronavirus 2 (SARS-CoV-2) continues to increase, so does the search for therapies to prevent its respiratory and multi-organ complications. Treatments under study have been classified into antiviral/repurposed ones, aiming to reduce viral uptake, and concomitant/adjunctive ones, aiming to minimise the risk of or to treat complications (Sanders et al., 2020). Despite the large number of proposed therapies, most drug trials have been negative: an increasing list of therapies including azithromycin, hydroxychloroquine, lopinavir/ritonavir, and tocilizumab have little or no impact on mortality or patient-relevant outcomes; whilst only corticosteroids and remdesivir have potential effects on mortality, mechanical ventilation, length of hospital stay, or duration of symptoms (Siemieniuk et al., 2020). Given the scarcity of available treatments, the search for medicines with putative effects to treat COVID-19 and minimize its complications is due to continue.

Activation of inflammatory and related cascades are part of innate immunity and crucial in the immune response against SARS-CoV-2. However, aggressive inflammatory response to SARS-CoV-2, known as cytokine release syndrome (CRS), has been recognized as a driving cause of high morbidity and mortality in COVID-19. In animal studies using mice, a direct disruption of catecholamine synthesis reduced cytokine release and offered protection against lethal complications of CRS (Staedtke et al., 2018). The same study found a similar protection from use of prazosin, an alpha-1 adrenergic receptor antagonist (alpha-1 blocker), to block catecholamines signalling. It has therefore been postulated that CRS may be prevented by targeting the catecholamine-cytokine axis.

Koenecke et al. (2021) investigated this hypothesis through a cohort study based primarily on US claims databases, comparing alpha-1 blocker users to non-users among patients hospitalized for acute respiratory distress or pneumonia. A companion paper by Rose et al. (2021) carried out a similar analysis for hospitalized COVID-19 patients in Veterans Health Administration hospitals. While they found the alpha-1 blocker users to have lower rates of progression to mechanical ventilation and/or mortality, such users vs. non-users comparisons are notoriously prone to systematic biases from residual confounding (Schneeweiss et al., 2007; Lund et al., 2015; Schuemie et al., 2019). In fact, Thomsen et al. (2021), another companion paper of theirs based on Danish cohorts hospitalized with pneumonia, found the difference in mortality to become closer to the null and substantially less significant when the comparator cohort is restricted to 5ARI users. Overall, given the complex and dynamic interplay between catecholamines and immune-inflammatory regulation, the net effect of inhibition of catecholamines on COVID-19 thus remains uncertain (Gubbi et al., 2020).

Currently, one randomized controlled trial is ongoing to study the efficacy of prazosin for SARS-CoV-2 infection (Thorlund et al., 2020). Clinical trials are inherently limited in sample size and patient representation, however. There is thus an urgent demand for further reliable and generalizable real-world evidence on the effect of alpha-1 blockers as a prophylaxis or treatment of COVID-19. In this article, we aimed to provide this much needed evidence by studying the association between the use of alpha-1 blockers and the susceptibility to COVID-19 disease (diagnosis), related hospitalization, and requirement of intensive services.

## Methods

### Study design

**FIGURE 1:**
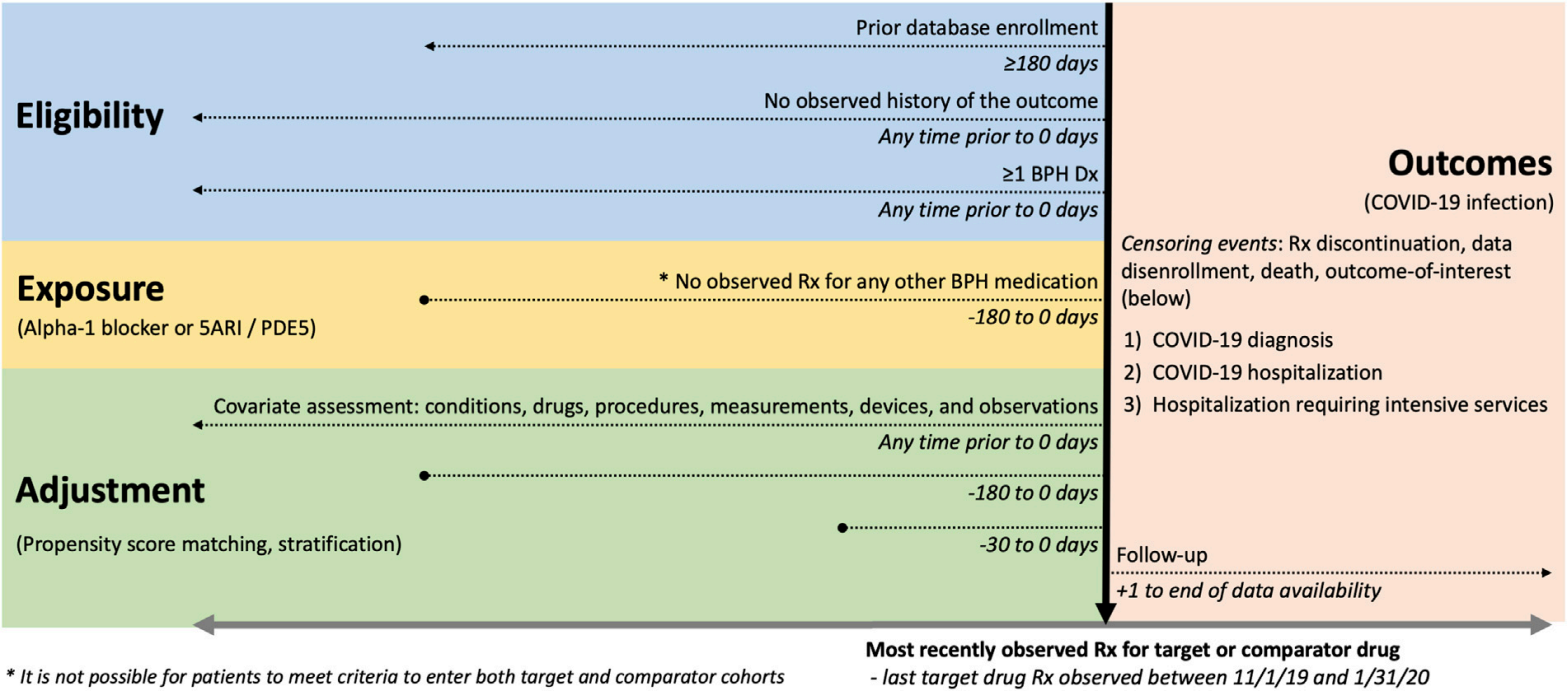
Alpha-1 blockers and susceptibility to COVID-19 study design schematic. We highlight eligibility criteria, exposure definitions, adjustment strategies, index date specification (horizontal black arrow) and outcome definitions and time-at-risk. Exposure involves prescriptions to drugs with RxNorm ingredients that map to medications indicated for treatment of BPH.

We conducted a prevalent-user active comparator cohort analysis across an internationally distributed network of databases. Our protocol is available at https://github.com/ohdsi-studies/Covid19SusceptibilityAlphaBlockers and registered in the EU PAS register (EUPAS36231). The study design described in this section is visually summarized in a schematic diagram of Figure 1.

### Data sources

We obtained routinely-collected electronic health records (EHRs) and claims data from Spain and the United States (US). All data sources had been previously mapped to the Observational Medical Outcomes Partnership (OMOP) Common Data Model (CDMv5) (Hripcsak et al., 2015). This enabled distributed network analyses without sharing patient-level data, whilst ensuring data provenance, by applying common analytical programmes across data partner centers. The included data sources are:

- Information System for Research in Primary Care (SIDIAP) database, covering approximately 80% of the population of Catalonia, Spain, or six million patients in number. SIDIAP contains data since 2006 from general practice EHRs linked to hospital admissions with information on diagnoses, prescriptions, laboratory tests, and lifestyle and sociodemographics and the central database of RT-PCR COVID-19 tests.
- US Department of Veterans Affairs (VA) database, covering approximately 12 million patients from 170 medical centers across the US and including administrative, clinical, laboratory, and pharmacy data repositories that are linked using unique patient identifiers (Lynch et al., 2019).
- Columbia University Irving Medical Center data warehouse (CUIMC) EHRs covering approximately six million patients from the New York-Presbyterian Hospital/Columbia University Irving Medical Center in the United States. CUIMC includes data on clinical diagnoses, prescriptions, laboratory tests, demographics, and diagnosis and test for COVID-19.
- IQVIA Open Claims, covering approximately 160 million patients in the US and providing pre-adjudicated health insurance claims at the anonymized patient-level. The data are collected from inpatient, outpatient, and pharmacy interactions that generates claims for the purpose of reimbursement.
- Optum^®^ De-Identified Clinformatics Data Mart Database—Date of Death (Optum DOD), covering approximately 86.8 million patients under private health insurance mostly in commercial plans but also in Medicare Advantage. Optum DOD includes data captured from administrative claims processed from inpatient and outpatient medical services and prescriptions as dispensed, as well as results for outpatient lab tests processed by large national lab vendors who participate in data exchange with Optum.
- Optum^®^ De-Identified COVID-19 Electronic Health Record (Optum EHR), covering approximately 1.73 million patients from a network of healthcare provider organizations across the US. Optum EHR includes data since 2007 on demographics, medications prescribed and administered, lab results, vital signs and other observable measurements, clinical and inpatient stay administrative data, diagnosis and test for COVID-19.

Each site obtained institutional review board approval, or confirmed the study to be exempt or deemed not human subjects research. At the time of this study, SIDIAP and IQVIA were last updated in July, 2020; CUIMC in August, 2020; and VA, Optum DOD, and Optum EHR in September, 2020.

### Cohort eligibility, study period and follow-up

Each cohort consisted of adult males aged 18 years or older who received at least one eligible prescription or dispensation for one of the study drugs between 1 November 2019 and 31 January 2020. Index date was set as the date of the last prescription in this time window. We required participants to be observable in their data source for at least 180 days prior to the index date and to have a recorded history of BPH at any time prior to or on the index date (Figure 1). Participants were then followed up until the earliest of: occurrence of an outcome; end of exposure (i.e., discontinuation of drug use); death; loss or deregistration from the database; or date of last data collection. The distribution of follow-up time and number of at-risk patients within each database is summarized in Supplementary Figures 1A,B.

### Exposures

We compared exposures to alpha-1 blockers (alfuzosin, doxazosin, prazosin, silodosin, tamsulosin, and terazosin) with exposures to other drug classes commonly indicated for treatment of BPH as active comparators. More precisely, the comparator consisted of dutasteride and finasteride (5ARI) and tadalafil (PDE5 inhibitor). The OMOP CDM concept IDs for these drug classes are provided in the supplement.

We restricted our analysis to subjects under monotherapy at cohort entry, excluding those who were exposed to alternative BPH treatments any time within 180 days prior to and including the index date. With the exception of our analysis on the Optum databases, we defined continuous drug exposures from the start of follow-up by grouping sequential prescriptions that have ≤30-day refill gaps between them. The end of exposure was defined as the end of the last prescription’s drug supply in such a sequence. In the Optum databases, it is difficult to identify periods of continuous drug exposure as prescriptions are not recorded consistently. We therefore used an intent-to-treat (ITT) type analysis, following patients until their record ends regardless of treatment persistence.

### Outcomes

We investigated three outcomes: 1) COVID-19 diagnosis; 2) COVID-19 hospitalization (inpatient visit with COVID-19 diagnosis during or up to 3 weeks prior to hospitalization); 3) COVID-19 hospitalization with intensive services (mechanical ventilation, tracheostomy, or extracorporeal membrane oxygenation). For all three outcomes, we required COVID-19 positive status determined through either test results or diagnostic codes. While administrative claims data only record administrations of COVID-19 tests and not their results, Kadri et al. (2020) has demonstrated the COVID-19 diagnosis code as a reliable proxy for a lab-confirmed case. The definitions of these outcomes are taken from the prior COVID-19 phenotype characterization study using OHDSI’s international database network (Burn et al., 2020; Morales et al., 2021; Prats-Uribe et al., 2021). We provide the full details of our cohort and outcome definitions in the supplement, as well as in the online protocol. We also provide an online data visualization tool (https://data.ohdsi.org/Covid19CharacterizationCharybdisDiagCovid/) to demonstrate the consistency and robustness of our outcome definitions across our databases.

### Study size

We included all patients meeting the eligibility criteria within each database and hence performed no *a priori* sample size calculation. Instead, we provided a minimum detectable rate ratio (MDRR) for each target-comparator-outcome triplet across each data source. MDRR is for achieving 5% type-1 error rate and 80% power.

### Statistical analyses

To adjust for measured confounding and improve covariate balance between comparison cohorts in a data-driven manner, we built large-scale PS models and fit them *via* regularized (ROSENBAUM and RUBIN, 1983; Tian et al., 2018). Our large-scale PS models consist of a broad range of pre-defined clinical covariates—including age, gender, race (US data), and other demographics, prior conditions, drug exposures, procedures, and health service utilization behaviors (Supplementary Table S4)—to allow for the most accurate prediction of treatment and balance cohorts across many characteristics. In particular, including the broad range of covariates gives us an opportunity to control for important confounders, such as baseline blood pressure, that may not have been directly captured by health databases but are associated with other surrogate variables present in the data (Schuemie et al., 2020). For computational efficiency, we excluded all features that occurred in fewer than 0.1% of patients within the target and comparator cohorts prior to PS model fitting.

In separate analyses, we stratified into 5 PS quintiles or variable-ratio matched patients by PS, and used Cox proportional hazards models to estimate hazard ratios (HRs) between alternative target and comparator treatments for the risk of each outcome in each data source. The regression conditioned on the PS strata/matching-unit with treatment allocation as the sole explanatory variable. We aggregated HR estimates across data sources to produce meta-analytic estimates using a random-effects meta-analysis with inverse-variance weights (DerSimonian and Laird, 1986). To study three outcomes in six data sources (plus one meta-analysis) using two PS-adjustment approaches, we generated 3 x (6 + 1) x 2 = 42 study effects.

Residual bias often remains in observational studies even after controlling for measured confounding through PS-adjustment (Schuemie et al., 2014; Schuemie et al., 2016). For each study, therefore, we conducted negative control outcome experiments, where the null hypothesis of no effect is believed to be true, using up to 118 controls identified through a data-rich algorithm (Voss et al., 2017) and then reviewed by clinicians. We provide the list of these negative control outcomes in Supplementary Table S3. The empirical null distributions from these experiments help us detect potential biases and under-conservative uncertainty estimates, thereby allowing us to debias point estimates and adjust confidence intervals (CI) as needed to achieve close to nominal operating characteristics; a more detailed description and demonstration of this calibration process (Schuemie et al., 2014; Schuemie et al., 2018) is provided in the supplement (see Supplementary Figure S2, for example). We calibrated each study effect HR estimate, its 95% CI and the *p*-value to reject the null hypothesis of no differential effect. We declared a HR as significantly different from no effect when the calibrated *p*-value is less than 0.05, without correcting for multiple testing.

Blinded to the results, clinicians, and epidemiologists evaluated study diagnostics for these treatment comparisons to assess if they were likely to yield unbiased estimates. The suite of diagnostics included 1) MDRR, 2) distributions of preference score, a transformation of PS that accounts for difference in exposure prevalences, (Walker et al., 2013) to evaluate empirical equipoise and population generalizability, 3) extensive patient characteristics to evaluate cohort balance before and after PS-adjustment, and 4) negative control calibration plots to assess residual bias. We defined target and comparator cohorts to stand in empirical equipoise if the majority of patients in both carry preference scores between 0.3 and 0.7 and to achieve sufficient balance if all after-adjustment baseline characteristics return absolute SMD < 0.1 (Austin, 2009).

### Study execution

We conducted this study using the open-source OHDSI CohortMethod R package (https://ohdsi.github.io/CohortMethod/) with large-scale analytics made possible through the Cyclops R package (Suchard et al., 2013). Start-to-finish open and executable source code is available at: https://github.com/ohdsi-studies/Covid19SusceptibilityAlphaBlockers. To promote transparency and facilitate sharing and exploration of the complete result set, an interactive web application (https://data.ohdsi.org/Covid19SusceptibilityAlphaBlockers/) serves up study diagnostics and results for all study effects.

## Results

### Baseline characteristics

**FIGURE 2:**
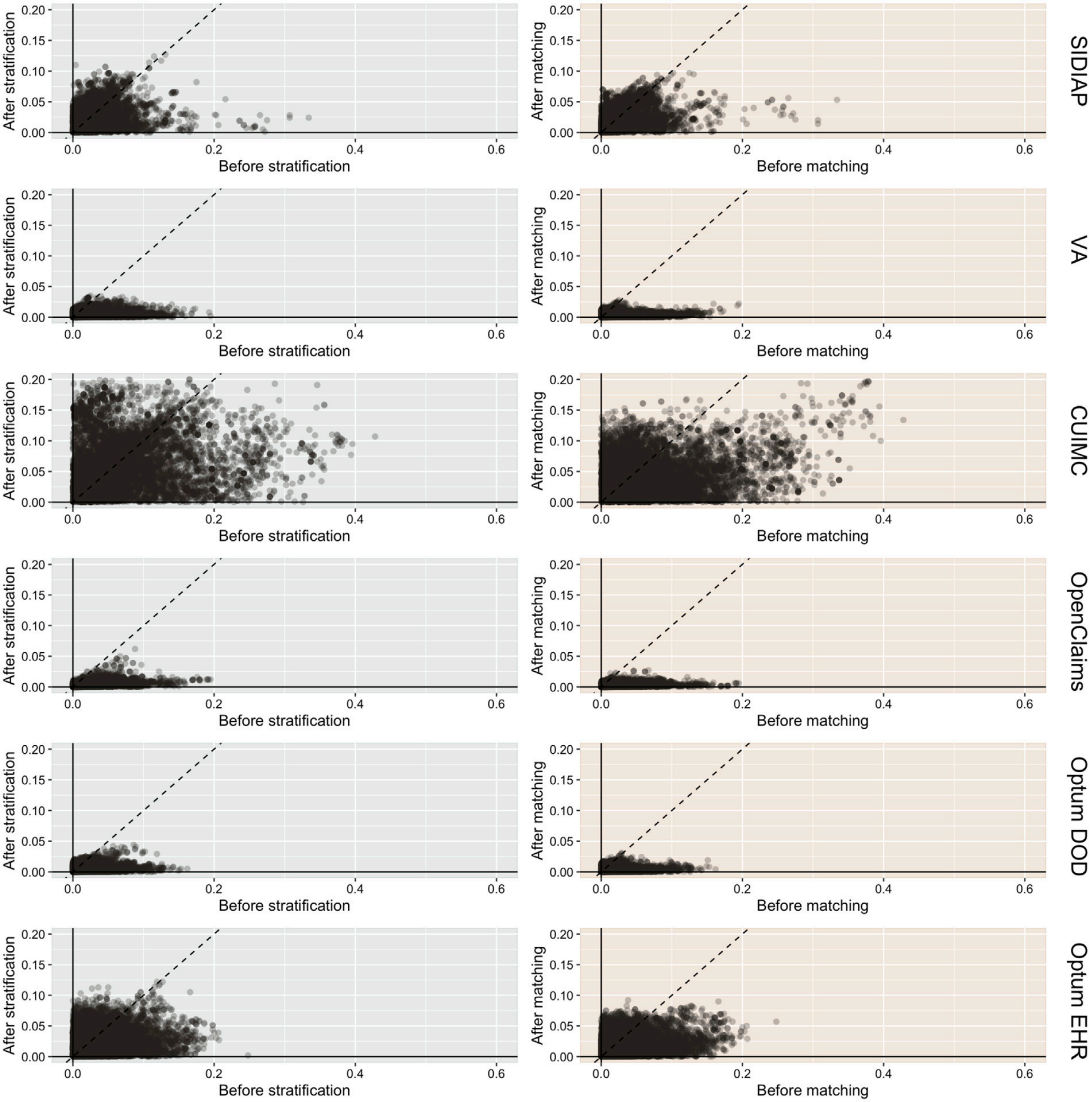
Cohort balance diagnostics comparing alpha-1 blocker and 5ARI/PDE5 prevalent users. We plot the absolute SMD of population proportions for all available patient characteristics (13,950 in SIDIAP, 81,436 in VA, 24,807 in CUIMC, 73,113 in OpenClaims, 79,184, in Optum DOD, 40,621 in Optum EHR) before and after propensity score stratification or matching across data sources. CUIMC fails study diagnostics under both stratification and matching since the absolute SMDs are not consistently <0.1. SIDIAP and Optum EHR fail study diagnostics under stratification only.

We found in total 2,628,170 users of alpha-1 blockers and 464,525 users of alternative BPH therapy—5-alpha reductase inhibitors and phosphodiesterase type 5 inhibitors (5ARI/PDE5)—with diagnosis of BPH, all of whom were included in our propensity score (PS) stratified analyses. For PS matched analysis, 2,426,765 (92.3%) and 463,113 (99.7%) of the subjects could be matched based on their baseline characteristics. Table 1 summarizes key socio-demographics, medical history and medicine use for alpha-1 blocker and active comparator users before and after PS stratification and matching. Table 1 focuses on OpenClaims and a selected subset of clinical covariates for an illustrative purpose, but Supplementary Table S1 provides detailed baseline characteristics for all participants in the six contributing data sources. On average, users of alpha-1 blockers were younger and healthier than users of active comparator medicines. For the most part, PS matching and stratification successfully reduced the differences in baseline characteristics to the negligible level of standardized mean differences (SMD) below 0.1 (Figure 2). Due to the relatively small cohort size, PS matching and stratification were less successful in CUIMC. A few covariates remained imbalanced (SMD > 0.1) after PS stratification in SIDIAP and Optum EHR. Except for these cases, no substantial differences in baseline characteristics remained after PS matching and stratification.

**TABLE 1:** Baseline patient characteristics for alpha-1 blocker and 5ARI/PDE5 user cohorts in the OpenClaims data source. For each target (T) and comparator (C) cohort, we report the proportion of initiators satisfying selected base-line characteristics and the standardized mean difference (SMD) between the two cohorts before and after stratification. The smaller SMDs after propensity score adjustment demonstrates improved balance between the two cohorts.

	Open claims					
Characterstics	Before stratification			After stratification		
	*N* = 1,995,594 (T) and 366,734 (C)					
	T (%)	C (%)	SMD	T (%)	C (%)	SMD
Age group						
<25	0.1	0.1	0.00	0.1	0.1	0.00
25–29	0.1	0.1	0.00	0.1	0.1	0.00
30–34	0.1	0.1	0.00	0.1	0.1	0.00
35–39	0.2	0.2	−0.01	0.2	0.2	0.00
40–44	0.4	0.4	0.01	0.4	0.4	0.01
45–49	1.2	0.8	0.04	1.2	1.0	0.01
50–54	3.1	1.8	0.08	2.9	2.7	0.01
55–59	7.0	4.1	0.13	6.6	6.2	0.02
60–64	12.2	8.1	0.14	11.6	11.3	0.01
65–69	17.4	13.9	0.10	16.8	16.9	0.00
70–74	19.2	19.2	0.00	19.2	19.1	0.00
75–79	16.7	19.2	−0.07	17.1	17.4	−0.01
80–84	17.1	24.1	−0.18	18.1	18.7	−0.02
85–89	5.3	7.8	−0.10	5.7	5.8	0.00
90–94						
95+						
Medical history: general						
Chronic liver disease	0.7	0.4	0.05	0.7	0.6	0.01
Chronic obstructive lung disease	7.0	5.4	0.07	6.8	6.7	0.00
Dementia	1.8	2.3	−0.03	1.9	1.9	0.00
Diabetes mellitus	19.3	16.0	0.09	18.8	18.6	0.00
Hyperlipidemia	29.8	28.9	0.02	29.7	29.6	0.00
Hypertensive disorder	38.2	34.8	0.07	37.7	37.6	0.00
Obesity	3.9	2.7	0.07	3.7	3.5	0.01
Renal impairment	11.2	9.7	0.05	11.1	10.8	0.01
Medical history: cardiovascular disease						
Cerebrovascular disease	3.3	3.2	0.05	11.1	10.8	0.01
Ischemic heart disease	4.0	3.6	0.03	4.0	4.0	0.00
Medical history: neoplasms						
Malignant neoplastic disease	11.1	9.7	0.04	10.9	10.7	0.01
Primary malignant neoplasm of prostate	4.8	2.9	0.10	4.6	4.2	0.02
Medication use						
Antiinflammatory and antirheumatic products	26.0	19.6	0.15	25.0	24.6	0.01
Antineoplastic agents	5.5	5.4	0.00	5.5	5.6	0.00
Antithrombotic agents	25.5	24.8	0.01	25.4	25.5	0.001
Drugs used in diabetes	26.1	21.5	0.11	25.5	25.4	0.00
Immunosuppressants	2.8	2.3	0.03	2.8	2.7	0.00

### Incidence rates of COVID-19-related outcomes

We report in Table 2 patient cohort size for each data source, in PS stratified and PS matched, and number of participants with each of the three outcomes of interest. In the PS-matched cohorts (excluding CUIMC, which did not achieve sufficient covariate balance), in total 6,319 alpha-1 blocker users of (275 in SIDIAP, 1,485 in VA, 4,351 in OpenClaims, 175 in Optum DOD, and 33 in Optum EHR) and 1,105 5ARI/PDE5 users (51 in SIDIAP, 236 in VA, 764 in OpenClaims, 47 in Optum DOD, and 7 in Optum EHR) were diagnosed with COVID-19. Incidence rates of COVID-19 diagnosis were 85.63/1,000 person-years amongst alpha-1 blockers, and 108.25/1,000 among 5ARI/PDE5 users in SIDIAP; 8.96 vs. 7.97/1,000 in VA; 5.62 vs. 4.78/1,000 in OpenClaims; 3.40 vs. 4.89/1,000 in Optum DOD; and 38.88 vs. 47.08/1,000 in Optum EHR. Similarly, a total of 3,108 alpha-1 blockers and 563 5ARI/PDE5 users were hospitalized with COVID-19. Incidence rates of hospital admission ranged from 2.29/1,000 in alpha-1 blocker users and 3.64/1,000 in 5ARI/PDE5 users in Optum DOD to 35.66 vs. 42.2/1,000 in SIDIAP. Finally, 110 (92 in VA, 18 in Optum DOD) alpha-1 blocker users and 18 (12 in VA, 6 in Optum DOD) 5ARI/PDE5 users received intensive services, with incidence rates of 0.55 vs. 0.40/1,000 respectively in VA, and 0.35 vs. 0.62/1,000 in Optum DOD. The data sources behind OpenClaims and SIDIAP do not contain information on intensive services, and Optum EHR and CUIMC have insufficiant sample sizes for this outcome.

**TABLE 2:** Populations and COVID-19 outcomes for alpha-1 blocker (T) and 5ARI/PDE5 (C) user cohorts. We report population size, total exposure time, outcome events (Covid diagnosis, hospitalization, and intensive services) and minimally detectable rate ratio (MDRR). MDRR is provided only for Covid diagnosis due to the space constraint. The database abbreviations are defined under the heading Data Sources in the Method section.

	Patients		Time (years)		Diagnosis		Hospital		Intensive		MDRR (diagnosis)
	T	C	T	C	T	C	T	C	T	C	
Stratified analysis											
SIDIAP	11,793	1,318	4,162	471	334	51	132	20	0	0	1.61
VA	360,802	54,723	189,564	29,642	1,854	236	636	96	111	12	1.20
CUIMC	2,414	582	338	84	27	<5	16	<5	0	0	4.53
Openclaims	1,995,594	366,734	817,994	160,225	4,809	767	2,621	407	0	0	1.11
Optum DOD	241,842	39,032	56,438	9,613	193	47	131	35	18	6	1.69
Optum EHR	15,275	2,136	1,031	149	50	7	32	5	<5	0	3.10
Matched analysis											
SIDIAP	8,994	1,315	3,211	471	275	51	115	20	0	0	1.59
VA	312,522	54,642	165,688	29,600	1,485	236	496	96	92	12	1.21
CUIMC	1,873	520	261	74	18	<5	11	<5	0	0	6.58
Openclaims	1,873,014	365,534	774,635	159,742	4,351	764	2,361	407	0	0	111
Optum DOD	218,032	38,988	51,451	9,602	175	47	118	35	18	6	1.69
Optum EHR	12,303	2,114	848	148	33	7	19	5	<5	0	3.50

### Hazard ratios of COVID-19-related outcomes

**FIGURE 3:**
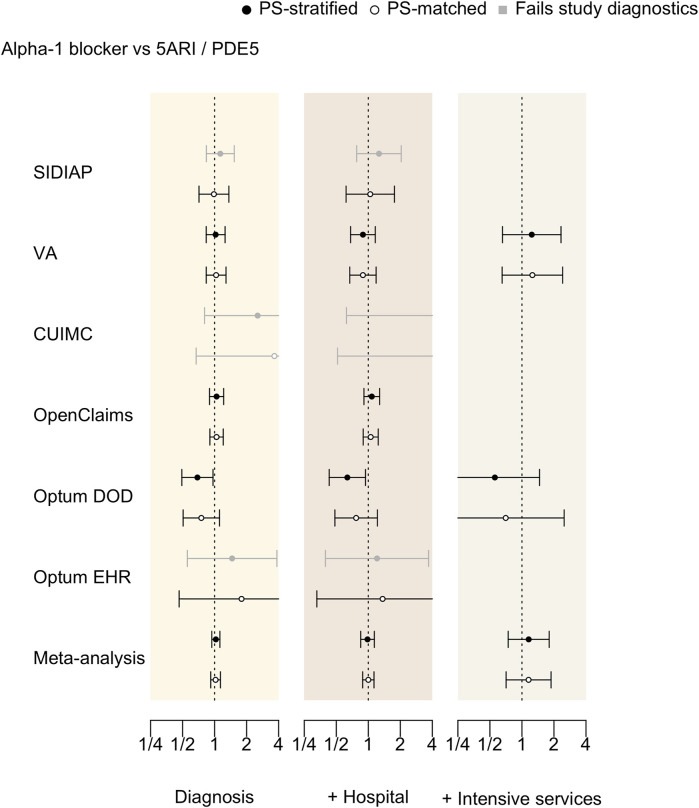
Hazard ratios of COVID-19 outcomes between alpha-1 blocker and 5ARI/PDE5 prevalent-use across data sources. The outcomes are COVID-19 diagnosis (Diagnosis), COVID-19 hospitalization (+Hospitalization), and COVID-19 hospitalization requiring intensive services (+Intensive services). We plot calibrated hazard ratios with black (PS-stratified) and white (PS-matched) circles along with their 95% confidence intervals. Grayed out entries do not pass study diagnostics.

Table 3 and Figure 3 present database-specific calibrated HRs for the risk of COVID-19 diagnosis, hospitalization, and intensive services under both PS stratified and matched analyses. The negative control experiment finds little evidence of residual confounding and thus calibrated and uncalibrated HRs are close to each other (Supplementary Table S2). Findings from both analyses are consistent with each other, but here we focus on the PS matched cases that passed the proposed diagnostics, which included all data sources except CUIMC. The risk of COVID-19 diagnosis did not differ between alpha-1 blocker and 5ARI/PDE5 users in any of the data sources, with PS-matched calibrated HR of 0.99 (95% CI 0.71–1.36) in SIDIAP, 1.03 (0.83–1.28) in VA, 1.04 (0.90–1.21) in OpenClaims, 0.75 (0.51–1.11) in Optum DOD, and 1.79 (0.46–6.92) in Optum EHR. The meta-analysis yields calibrated HR of 1.02 (95%CI 0.92–1.13) for COVID-19 diagnosis.

**TABLE 3:** Hazard ratios of COVID-19 diagnosis, hospitalization, and intensive services for alpha-1 blocker and 5ARI/PDE5 prevalent-use. We report calibrated hazard ratios (HRs) and their 95% confidence intervals (CIs) and calibrated *p*-value (*p*), with PS stratification or matching and across data sources. Grayed out entries do not pass study diagnostics and are excluded from the meta-analysis.

	PS-stratified			PS-matched		
	HR	PI CI	*p*	HR	PI CI	*p*
Diagnosis						
SIDIAP	1.13	(0.84–1.53)	0.54	0.99	(0.71–1.36)	0.64
VA	1.02	(0.83–1.26)	0.81	1.03	(0.83–1.28)	0.76
CUIMC	2.54	(0.80–8.01)	0.14	3.65	(0.67–19.9)	0.15
Openclaims	1.04	(0.90–1.22)	0.58	1.04	(0.90–1.21	0.56
Optum DOD	0.69	(0.49–0.97)	0.03	0.75	(0.51–1.11)	0.15
Optum EHR	1.46	(0.55–3.85)	0.43	1.79	(0.46–6.92)	0.39
Meta-analysis	1.03	(0.94–1.12)	0.54	1.02	(0.92–1.13)	0.68
+ Hospitalization						
SIDIAP	1.26	(0.78–2.04)	0.43	1.04	(0.62–1.76)	0.74
VA	0.89	(0.68–1.16)	0.40	0.89	(0.67–1.19)	0.43
CUIMC	6.33	(0.62–64.3)	0.13	5.92	(0.51–68.2)	0.16
Openclaims	1.08	(0.91–1.28)	0.38	1.05	(0.90–1.24)	0.53
Optum DOD	0.64	(0.43–0.94)	0.02	0.77	(0.49–1.22)	0.26
Optum EHR	1.21	(0.40–3.69)	0.74	1.36	(0.33–5.66)	0.67
Meta-analysis	0.98	(0.85–1.14)	0.83	1.00	(0.89–1.13)	0.94
+ Intensive services						
SIDIAP	NA	NA	NA	NA	NA	NA
VA	1.24	(0.66–2.33)	0.51	1.25	(0.65–2.41)	0.50
CUIMC	NA	NA	NA	NA	NA	NA
Openclaims	NA	NA	NA	NA	NA	NA
Optum DOD	0.56	(0.21–1.46)	0.23	0.70	(0.20–2.49)	0.59
Optum EHR	NA	NA	NA	NA	NA	NA
Meta-analysis	1.16	(0.74–1.80)	0.52	1.15	(0.71–1.88)	5.56

For COVID-19 hospitalization, PS-matched analyses again found no differential risks according to drug use in any of the contributing data sources, with calibrated HRs of 1.04 (0.62–1.76) in SIDIAP, 0.89 (0.67–1.19) in VA, 1.05 (0.90–1.24) in OpenClaims, 0.77 (0.49–1.22) in Optum DOD, 1.36 (0.33–5.66) in Optum EHR, and 1.00 (0.89–1.13) in the meta-analysis.

For COVID-19 hospitalization requiring intensive services, only VA and Optum DOD passed diagnostics, with PS-matched calibrated HRs 1.25 (0.65–2.41) and 0.70 (0.20–2.49) respectively. Meta-analytic HR was 1.15 (0.71–1.88).

Out of the 118 pre-selected negative control outcomes, we used from 22 to 101 of them within each data source as we did not find sufficient numbers of events for the rest of negative controls.

## Discussion

In this international cohort study following more than 3.1 million men with BPH, we observed no association between alpha-1 blocker use and the risk of COVID-19 diagnosis, hospitalization, or hospitalization requiring intensive services. These findings bear out from 6 large real-world data sources, including out- and in-patient electronic medical records and health claims data from the US and Spain.

To our knowledge, Koenecke et al. (2021), along with their companion papers Thomsen et al. (2021) and Rose et al. (2021), are the only existing epidemiological studies exploring the potential association between alpha-1 blockers use and disease progression induced by lower respiratory tract infection. In this retrospective analysis of patients with acute respiratory distress or pneumonia, previous users of alpha-1 blockers—as compared to non-users—demonstrated lower risks of progression to ventilation and/or death. Given the very limited sets of covariates considered in their analyses, however, there is serious concern for substantial residual confounding. Moreover, healthy adherer bias (Hollestein et al., 2015) could have been introduced by their choice to include only patients who were prescribed alpha-1 blockers and had a medication possession ratio ≥50% in the year before the index date. Persistent alpha-1 blocker users likely have healthier lifestyles, such as sensible diet and regular exercise, than people who do not use or adhere to the medication. Such healthy adherer bias may have distorted their estimates towards the protective.

In our study, we selected 5ARI and PDE5 inhibitors as active comparators to minimize confounding by indication, which often leads to informed presence biases, channeling biases, as well as an array of other unexpected biases in observational health data (Bosco et al., 2010; Brookhart et al., 2010; Goldstein et al., 2016; Weinstein et al., 2017). We then confined our study population to patients with prior diagnosis of BPH in order to account for the fact that these drugs do not share all indications. In addition to the active comparator selection, we applied large-scale propensity score models involving tens of thousands of clinical covariates, thereby balancing a broad range of baseline patient characteristics. The conventional approach to adjust only for a small number of pre-selected covariates would have left many baseline characteristics imbalanced, which is particularly problematic when clinicians’ knowledge regarding this novel disease is limited.

The initial rationale that the disruption of catecholamine loop reduces CRS resulting from bacterial and non-bacterial causes was based on an animal study published in 2018 (Staedtke et al., 2018). After the pandemic began, the authors from this study postulated that prophylactic use of alpha-1 blockers might decrease the risk of progression to life-threatening complication among COVID-19 patients (Konig et al., 2020). This hypothesis was formed when three pieces of information were stacked: first, severe COVID-19 patients were often accompanied by a significant elevation of cytokines such as interleukin-6, interleukin-10 and tumour necrosis factor *α*. Second, catecholamines augmented the production of those cytokines *in vitro* and mice. Last, blockade of the alpha-1 adrenergic receptor (target of catecholamines) suppresses hyperinflammatory state in the context of bacterial infections.

However, we argue that preventing severe illness from the SARS-CoV-2 by targeting catecholamine-cytokine axis with the use of alpha-1 blockers is far more complicated and multifaceted. For example, use of alpha-1 blockers might increase the release of catecholamines through the negative feedback loop, (Zuber et al., 2011) which in turn counteracts the benefit resulting from inhibition of alpha-1 adrenergic receptors. One the other hand, the surge of cytokines is more likely to be a consequence of patients responding aggressively to an infection with SARS-CoV-2. The reduction of cytokines by deactivation of alpha-1 adrenergic receptors thus may be insufficient to cut off, once triggered, the cascade of CRS involving a series of white blood cells and signaling molecules.

### Strengths and limitations

This open science study comes with certain limitations, but also with unique strengths by virtue of our access to an international network of standardized databases. Below, we discuss potential limitations one by one, as well as ways through which our study addresses them to the extent practically possible.

First, the study only partially addresses the question of whether alpha-1 blockers alleviate the disease progression of COVID-19 as postulated by Konig et al. (2020). With COVID-19 being an emergent disease, the number of in-patient COVID-19 cases was rather small during the studied period even in the extensive network of databases we have access to. We thus determined the number to be insufficient for us to directly estimate the effectiveness against the disease progression in a scientifically meaningful manner. Instead, we attempted to investigate the question under the hypothesis that, if alpha-1 blockers were indeed protective against severe COVID-19 symptoms, we should see a negative association between the prevalent use of alpha-1 blockers and COVID-19 related outcomes such as hospitalization. Even COVID-19 diagnosis alone could be indicative of relatively severe symptoms since patients are otherwise unlikely to seek interactions with healthcare systems.

Second, we used a prevalent-user cohort design since there are so few patients initiating BPH therapies during and immediately preceding the pandemic that a new-user design is infeasible. A prevalent-user design is susceptible to potential bias due to time-varying hazards and/or inclusion of treatment effect mediators in the adjustment. In particular, our finding does not eliminate the possibility that the incident use—but not prevalent use—of alpha-1 blockers protects against COVID-19.

Third, while we have taken great care in identifying prescriptions of the study drugs, patients’ adherence to prescribed drugs cannot be determined from secondary observational health data. Presence (or absence) of prescription records in EHR or claims databases does not guarantee that the patient was in fact taking (or not taking) the prescribed drug. We mitigate this problem, however, by applying an active comparator for which we expect the misclassification rate to be similar to the target drug. This way, uncertainty in the drug usages would affect the magnitude of hazard ratios, but not its direction. By a similar mechanism, the use of active comparators also mitigates the issue of potential under-diagnosis/reporting of COVID-19; as long as its rate is similar in both cohorts, under-diagnosis/reporting is likely to only affect the magnitude of the hazard ratio, but not its direction.

Fourth, another important limitation is under-diagnosis or under-reporting of COVID-19. The limited test availability during the early months of the pandemic further contributes to this issue. We alleviated this issue by using a definition of COVID-19 based on broad data sources, including clinical diagnosis and/or PCR test data. However, this strategy still cannot account for many infected patients who likely remain asymptomatic or do not seek health care services. The issue is further exacerbated by the fact that the diagnosis and reporting of COVID-19 related data vary significantly over different sites and at different time points in the pandemic. For example, the data sources behind OpenClaims and SIDIAP do not contain information on intensive services. While no observational study of a potential COVID-19 treatment is immune to these caveats, we believe that our approach—with consistent applications of the same design and analysis over an international network of observational databases—provides some of the most reliable real-world evidence. We found some variation in the estimates across our databases but not at a statistically significant level, giving us confidence in our study design choice and overall conclusion. Single-center observational studies, on the other hand, have no ways to assess whether their findings would hold under different data sources.

Fifth, the use of 5ARI/PDE5 as active comparators—a necessity for properly dealing with confounding by indication (Schneeweiss et al., 2007; Bosco et al., 2010; Lund et al., 2015; Schuemie et al., 2019)—means that, technically, our result only shows a lack of therapeutic effects from alpha-1 blockers relative to 5ARI/PDE5. In other words, there remains an arguably small possibility that alpha-1 blockers are actually beneficial (or harmful) in treating COVID-19; just no more or less so than 5ARI/PDE5.

Finally, we conducted this study among adult men with BPH to guard against confounding by indication. Thus, these findings may not generalize to wider populations. However, older male patients constitute a particularly relevant high-risk subpopulation, accounting for a substantial portion of the severe cases of COVID-19. Also, there is currently no evidence that the pathophysiology of BPH modifies the effect of alpha-1 blockers on COVID-19.

## Conclusion

Our findings do not support prophylactic use of alpha-1 blockers to decrease the risk of COVID-19 infection and progression among, e.g., people with suspected exposure to the virus. Further research is needed to determine the potential therapeutic effect of alpha-1 blocker initiation on people who are recently infected with SARS-CoV-2.

## Data Availability

All data sources had been previously mapped to the Observational Medical Outcomes Partnership (OMOP) Common Data Model (CDMv5) (Hripcsak et al. 2015). This enabled distributed network analyses without sharing patient-level data, whilst ensuring data provenance, by applying common analytical programmes across data partner centers. We conducted this study using the open-source OHDSI CohortMethod R package (https://ohdsi.github.io/CohortMethod/) with large-scale analytics made possible through the Cyclops R package (Suchard et al. 2013). Start-to-finish open and executable source code is available at: https://tgithub.com/ohdsi-studies/Covid19SusceptibilityAlphaBlockers. To promote transparency and facilitate sharing and exploration of the complete result set, an interactive web application (https://data.ohdsi.org/Covid19SusceptibilityAlphaBlockers/) serves up study diagnostics and results for all study effects.
