# Supplementary material for: International cohort study indicates no association between alpha-1 blockers and susceptibility to COVID-19 in benign prostatic hyperplasia patients
Source: Front Pharmacol. 2022 Sep 14;13:945592. doi: 10.3389/fphar.2022.945592 (PMC9518954; doi:10.3389/fphar.2022.945592)
Supplement: Supplementary file 1 [file Presentation1.pdf]

Supplement to "Use of alpha-1 blockers and susceptibility to COVID-19 in patients with benign prostate hyperplasia: an international cohort study"

Study period and follow-up time

As described in the main text, we indexed our cohorts at the last prescription of the drugs between November 1st, 2019 and January 31st and followed participants until the earliest of: occurrence of an outcome; end of exposure; death; loss or deregistration from the database; or date of last data collection. We summarized the distribution of the follow-up times by calculating how many patients remain at risk after 50, 100, 150, … days after their index dates within each database. We include tables of this information alongside Kaplan-Meier plots in Supplementary Figure 1 and 2.

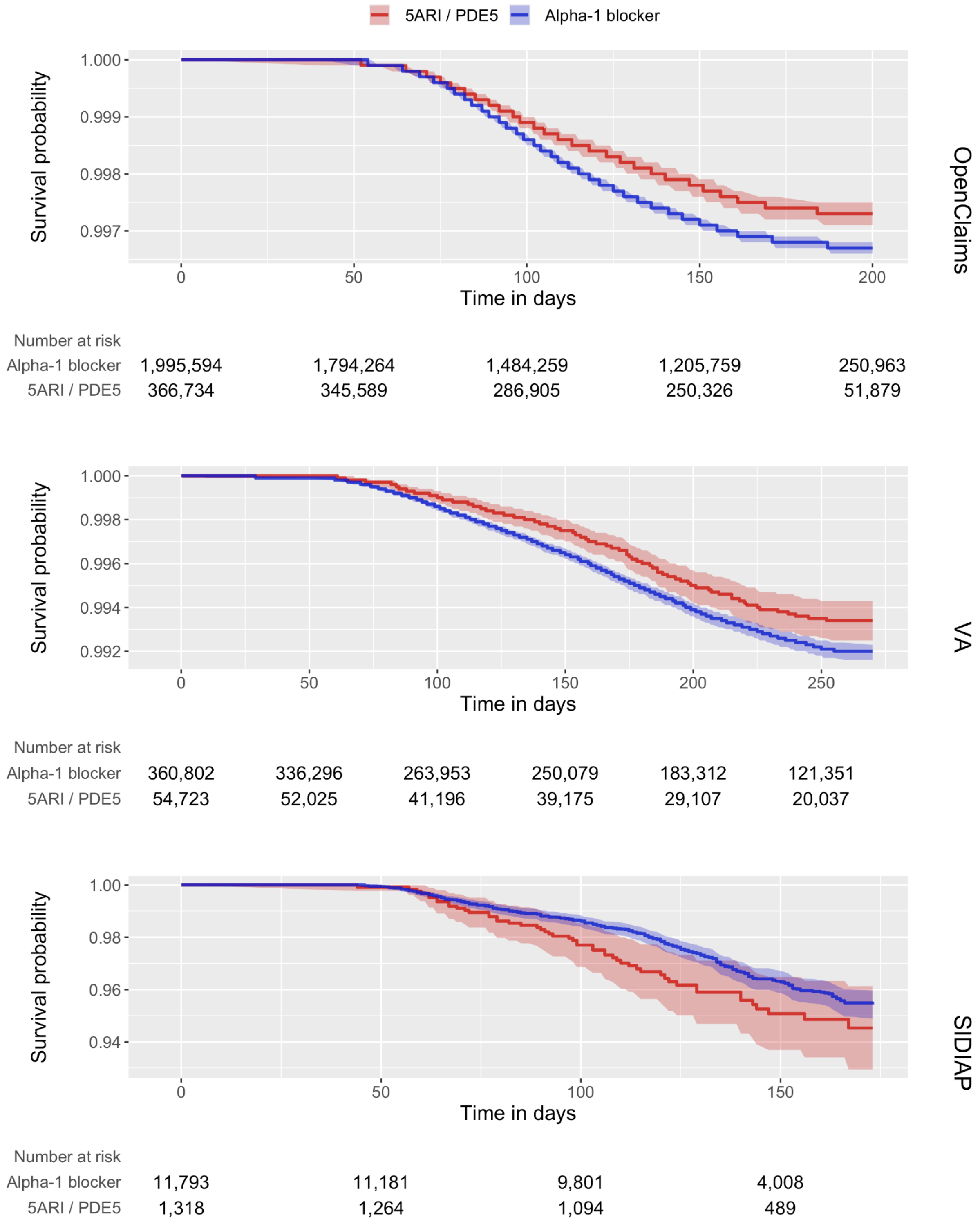

Supplementary Figure 1(a): Kaplan-Meier plots for time until COVID-19 diagnosis in the three databases with greater statistical power in terms of estimated MDRRs (OpenClaims, VA, and SIDIAP). Shown beneath each plot are the numbers of at-risk patients in each cohort at various points in time.

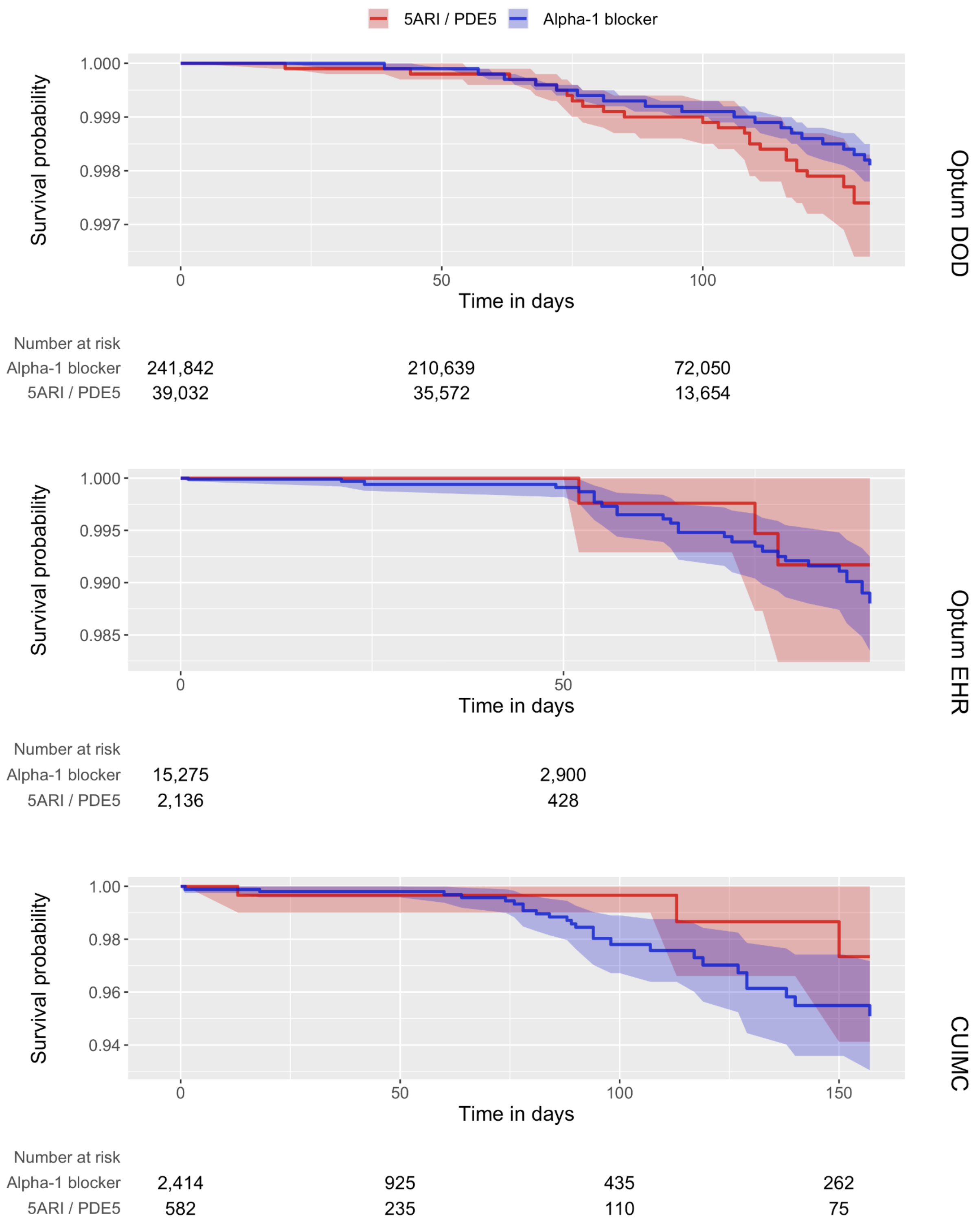

Supplementary Figure 1(b): Kaplan-Meier plots for time until COVID-19 diagnosis in the three databases with lesser statistical power in terms of estimated MDRRs (Optum DOD, Optum EHR, and CUIMC). Shown beneath each plot are the numbers of at-risk patients in each cohort at various points in time.

Patient characteristics pre- and post-stratification

Supplementary Table 1: Baseline patient characteristics for Alpha-1 blocker (T) and 5ARI/PDE5 (C) prevalent-use in the SIDIAP, VA, CUIMC, OpenClaims, Optum DOD, and Optum EHR data sources. We report the proportion of use satisfying selected based-line characteristics and the standardized difference of population proportions (SDf) before and after stratification. Less extreme SDf through stratification suggest improved balance between patient cohorts through propensity score adjustment.

Characteristic	SIDIAP						VA						CUIMC					
	Before stratification N = 11,793 (T)			After stratification and 1,318 (C)			Before stratification N = 360,802 (T)			After stratification and 54,723 (C)			Before stratification N = 2,414 (T)			After stratification and 582 (C)		
	T (%)	C (%)	SDf	T (%)	C (%)	SDf	T (%)	C (%)	SDf	T (%)	C (%)	SDf	T (%)	C (%)	SDf	T (%)	C (%)	SDf
Age group																		
<25																		
25-29	0.0	<0.4		0.0	<0.3		0.0	0.0		0.0	0.0							
30-34	0.0	<0.4	-0.05	0.0	<0.3	-0.06	0.0	0.0	0.02	0.0	0.0	0.01	<0.2	0.0		<0.2	0.0	
35-39	0.0	<0.4	-0.04	0.0	<0.3	-0.04	0.1	0.0	0.04	0.1	0.0	0.03	0.2	<0.9	0.01	0.2	<0.9	0.04
40-44	0.3	0.0		0.3	0.0		0.3	0.1	0.05	0.3	0.1	0.03	0.5	<0.9	0.02	0.5	<0.9	0.04
45-49	1.1	0.5	0.06	1.1	0.7	0.04	0.8	0.3	0.07	0.7	0.6	0.02	1.1	<0.9	0.07	1.2	<0.9	0.12
50-54	3.2	0.8	0.17	3.0	1.7	0.08	2.1	0.7	0.11	1.9	1.7	0.01	3.0	2.1	0.06	3.0	1.9	0.08
55-59	7.5	3.0	0.21	7.1	7.4	-0.01	4.5	2.0	0.14	4.1	4.1	0.00	6.3	3.4	0.13	5.9	5.7	0.01
60-64	12.2	6.0	0.22	11.5	13.3	-0.05	8.5	4.7	0.16	8.0	8.2	0.00	12.3	3.6	0.32	10.5	11.8	-0.04
65-69	16.3	13.4	0.08	16.0	15.5	0.01	14.4	10.0	0.13	13.7	14.0	-0.01	16.2	12.2	0.12	15.5	14.3	0.03
70-74	19.2	16.8	0.06	19.0	19.4	-0.01	31.9	28.1	0.08	31.3	31.8	-0.01	18.6	19.6	-0.02	18.6	20.0	-0.04
75-79	17.7	19.2	-0.04	17.8	17.7	0.00	15.8	17.1	-0.04	16.0	15.7	0.01	16.1	18.9	-0.07	16.4	15.4	0.03
80-84	11.7	17.8	-0.17	12.6	11.7	0.03	9.1	12.9	-0.12	9.6	9.4	0.01	13.6	18.0	-0.12	14.5	15.2	-0.02
85-89	7.6	13.9	-0.20	8.3	7.6	0.03	7.8	13.8	-0.19	8.6	8.4	0.01	7.0	12.5	-0.18	8.1	9.1	-0.04
90-94	2.6	6.9	-0.20	3.0	3.5	-0.03	3.6	7.5	-0.17	4.1	4.3	-0.01	3.9	7.5	-0.16	4.3	5.0	-0.03
95+	0.4	1.1	-0.08	0.4	0.9	-0.06	1.1	2.5	-0.11	1.3	1.4	-0.02	0.9	1.0	-0.01	1.0	<0.9	0.02
Gender: female																		
Medical history: General																		
Acute respiratory disease	8.7	9.6	-0.03	8.8	8.7	0.00	6.6	5.3	0.06	6.4	6.3	0.00	7.2	3.9	0.14	6.6	6.3	0.01
Attention deficit hyperactivity disorder	0.1	<0.4	-0.03	0.1	0.5	-0.08	0.4	0.2	0.03	0.4	0.4	0.00	<0.2	<0.9	0.00	<0.2	<0.9	-0.02
Chronic liver disease	1.5	0.9	0.06	1.5	0.8	0.06	2.6	1.5	0.08	2.5	2.3	0.01	2.2	<0.9	0.13	1.9	2.0	0.00
Chronic obstructive lung disease	12.6	15.8	-0.09	12.9	13.8	-0.02	15.7	14.3	0.04	15.6	15.6	0.00	7.6	3.1	0.20	6.8	5.8	0.04
Crohn's disease	0.2	0.5	-0.06	0.2	0.6	-0.06	0.2	0.2	0.01	0.2	0.2	0.00	0.2	<0.9	0.02	0.2	<0.9	0.04
Dementia	2.8	5.1	-0.12	2.9	3.3	-0.02	3.9	6.0	-0.10	4.1	4.3	-0.01	4.7	3.3	0.07	4.5	5.3	-0.03
Depressive disorder	9.3	9.8	-0.02	9.4	9.4	0.00	18.8	14.2	0.13	18.2	18.0	0.01	8.4	4.3	0.17	7.7	5.3	0.10
Diabetes mellitus	24.1	23.8	0.01	24.3	23.9	0.01	32.8	31.0	0.04	32.5	32.8	0.00	24.6	15.1	0.24	22.4	22.8	-0.01
Gastroesophageal reflux disease	10.3	11.7	-0.04	10.4	12.2	-0.06	18.9	17.7	0.03	18.7	18.7	0.00	11.6	8.4	0.10	10.5	11.4	-0.03
Gastrointestinal hemorrhage	0.9	0.5	0.06	0.9	0.4	0.07	1.3	1.0	0.03	1.2	1.2	0.00	2.4	2.1	0.02	2.2	2.9	-0.04
Human immunodeficiency virus infection	0.3	0.4	-0.01	0.3	0.6	-0.04	0.5	0.4	0.01	0.5	0.5	0.00	2.7	1.4	0.09	2.5	4.1	-0.09
Hyperlipidemia	24.7	26.6	-0.04	24.8	25.2	-0.01	48.7	48.3	0.01	48.6	48.3	0.01	41.6	33.8	0.16	39.9	44.7	-0.10
Hypertensive disorder	55.0	58.8	-0.08	55.6	53.0	0.05	58.6	57.0	0.03	58.4	58.1	0.01	50.7	36.4	0.29	47.5	51.1	-0.07
Lesion of liver	1.1	0.8	0.04	1.1	1.0	0.01	2.1	1.3	0.06	2.0	1.8	0.01	2.9	<0.9	0.17	2.5	2.4	0.01
Obesity	28.6	27.2	0.03	28.5	28.1	0.01	12.3	8.8	0.11	11.8	11.7	0.00	7.3	4.3	0.13	6.7	6.2	0.02
Osteoarthritis	29.1	33.9	-0.10	29.6	29.0	0.01	18.3	16.5	0.05	18.1	18.1	0.00	16.2	11.7	0.13	14.8	13.8	0.03
Pneumonia	1.0	1.1	0.00	1.0	1.1	-0.01	2.2	2.0	0.01	2.1	2.1	0.00	6.0	2.7	0.16	5.3	3.9	0.07
Psoriasis	4.4	3.4	0.05	4.3	2.9	0.08	1.1	1.1	0.00	1.1	1.3	-0.01	1.1	0.9	0.03	1.0	1.2	-0.02
Renal impairment	14.4	18.7	-0.11	14.9	13.4	0.04	14.2	14.5	-0.01	14.2	14.2	0.00	22.4	13.9	0.22	20.4	21.1	-0.02
Rheumatoid arthritis	0.2	0.5	-0.05	0.2	0.5	-0.04	1.0	1.0	0.00	0.9	1.0	0.00	0.4	<0.9	0.01	0.4	<0.9	0.03
Schizophrenia	0.4	0.5	-0.03	0.4	0.6	-0.04	1.0	0.8	0.02	1.0	1.1	-0.01	0.4	<0.9	0.04	0.4	1.5	-0.12
Ulcerative colitis	0.6	0.8	-0.03	0.5	0.9	-0.04	0.4	0.4	0.00	0.4	0.4	-0.01	0.2	<0.9	0.02	<0.2	1.5	-0.15
Urinary tract infectious disease	5.7	5.9	-0.01	5.8	6.0	-0.01	3.0	3.1	0.00	3.0	3.1	0.00	8.8	7.2	0.06	8.3	9.7	-0.05
Viral hepatitis C	0.9	0.6	0.04	0.9	0.6	0.04	1.6	0.8	0.07	1.5	1.3	0.02	1.0	<0.9	0.03	0.9	2.0	-0.10
Visual system disorder	40.9	45.2	-0.09	41.5	40.9	0.01	34.1	33.8	0.00	34.0	34.4	-0.01	15.2	15.1	0.00	14.7	17.1	-0.07

(Continued)

Characteristic	SIDIAP						VA						CUIMC					
	Before stratification N = 11,793 (T)			After stratification and 1,318 (C)			Before stratification N = 360,802 (T)			After stratification and 54,723 (C)			Before stratification N = 2,414 (T)			After stratification and 582 (C)		
	T (%)	C (%)	SDf	T (%)	C (%)	SDf	T (%)	C (%)	SDf	T (%)	C (%)	SDf	T (%)	C (%)	SDf	T (%)	C (%)	SDf
Medical history: Cardiovascular disease																		
Atrial fibrillation	9.0	14.3	-0.17	9.5	9.7	0.00	11.4	14.4	-0.09	11.8	11.9	0.00	14.2	17.5	-0.09	14.8	16.7	-0.05
Cerebrovascular disease	3.0	3.9	-0.05	3.1	3.1	0.00	4.0	4.2	-0.01	4.0	3.9	0.00	8.4	6.7	0.06	7.8	9.9	-0.07
Coronary arteriosclerosis	0.0	<0.4	-0.02	0.0	<0.3	-0.01	17.8	20.1	-0.06	18.1	18.1	0.00	23.4	21.6	0.04	22.7	26.0	-0.08
Heart disease	34.4	42.9	-0.17	35.4	34.8	0.01	33.5	37.6	-0.08	34.1	34.0	0.00	43.4	39.5	0.08	42.5	44.6	-0.04
Heart failure	4.2	8.3	-0.17	4.5	5.0	-0.02	7.7	8.6	-0.03	7.8	7.9	0.00	13.3	10.8	0.07	12.6	14.4	-0.05
Ischemic heart disease	10.6	12.8	-0.07	10.9	10.7	0.00	6.4	6.6	-0.01	6.5	6.4	0.00	9.2	7.2	0.07	8.4	11.8	-0.11
Peripheral vascular disease	5.4	6.2	-0.03	5.5	5.5	0.00	5.4	5.6	-0.01	5.5	5.4	0.00	5.9	5.3	0.03	5.5	8.9	-0.13
Pulmonary embolism	0.7	1.6	-0.08	0.8	1.7	-0.09	0.7	0.7	0.00	0.7	0.7	0.00	1.2	1.2	0.00	1.0	1.7	-0.05
Venous thrombosis	1.3	1.2	0.01	1.3	1.4	-0.01	1.3	1.4	0.00	1.3	1.4	-0.01	2.6	<0.9	0.17	2.4	<0.9	0.15
Medical history: Neoplasms																		
Hematologic neoplasm	0.8	0.8	0.00	0.8	0.6	0.03	1.6	1.7	-0.01	1.6	1.6	0.00	3.5	2.2	0.07	3.3	3.1	0.01
Malignant lymphoma	0.7	0.5	0.03	0.7	0.3	0.05	0.9	1.0	-0.01	1.0	0.9	0.00	2.4	2.6	-0.01	2.3	3.6	-0.08
Malignant neoplasm of anorectum	0.6	0.5	0.02	0.6	0.5	0.01	0.2	0.1	0.01	0.2	0.1	0.01	0.3	0.0		0.3	0.0	
Malignant neoplastic disease	18.4	19.4	-0.03	18.5	17.5	0.03	12.9	11.5	0.04	12.7	12.3	0.01	20.9	15.6	0.14	20.0	19.8	0.01
Malignant tumor of breast	0.1	<0.4	-0.01	0.1	<0.3	0.01	0.0	0.0	0.00	0.0	0.0	0.00	<0.2	<0.9	-0.03	<0.2	<0.9	-0.08
Malignant tumor of colon	2.2	2.2	0.00	2.2	1.9	0.02	0.4	0.4	0.00	0.4	0.4	0.00	0.9	<0.9	0.03	0.8	1.0	-0.01
Malignant tumor of lung	0.2	<0.4	-0.01	0.2	<0.3	-0.01	0.8	0.7	0.01	0.8	0.7	0.00	1.4	0.0		1.3	0.0	
Malignant tumor of urinary bladder	2.8	3.8	-0.05	2.9	2.3	0.04	0.9	1.0	0.00	0.9	1.0	0.00	2.2	2.9	-0.04	2.4	3.6	-0.07
Primary malignant neoplasm of prostate	4.1	1.7	0.15	3.9	2.7	0.07	5.2	3.2	0.10	4.9	4.6	0.01	7.2	4.8	0.10	7.0	5.0	0.09
Medication use																		
Antibacterials for systemic use	28.5	27.8	0.01	28.5	28.1	0.01	24.9	22.2	0.06	24.5	24.4	0.00	42.9	34.8	0.16	41.4	45.5	-0.08
Antidepressants	15.4	18.3	-0.08	15.6	15.2	0.01	37.1	29.9	0.15	36.1	35.9	0.00	19.1	15.6	0.09	18.3	19.1	-0.02
Antiepileptics	10.0	9.1	0.03	10.1	9.6	0.02	29.1	24.1	0.11	28.5	28.1	0.01	20.2	12.5	0.21	18.8	18.6	0.00
Antiinflammatory and antirheumatic products	22.0	17.6	0.11	21.5	21.5	0.00	43.6	37.2	0.13	42.7	42.8	0.00	20.0	15.1	0.13	18.8	20.3	-0.04
Antineoplastic agents	1.2	1.1	0.01	1.2	0.9	0.03	4.8	4.5	0.01	4.8	4.7	0.00	5.0	3.3	0.09	4.5	6.1	-0.07
Antipsoriatics	2.1	1.9	0.02	2.1	1.6	0.04	1.4	1.4	0.00	1.4	1.4	0.00	1.9	0.9	0.09	1.7	<0.9	0.11
Antithrombotic agents	37.7	45.2	-0.15	38.7	37.2	0.03	39.6	40.1	-0.01	39.7	39.7	0.00	42.1	29.3	0.27	39.2	41.1	-0.04
Drugs for acid related disorders	47.7	48.9	-0.03	47.9	45.0	0.06	48.3	44.9	0.07	47.8	47.8	0.00	35.4	27.4	0.17	33.3	36.8	-0.07
Drugs for obstructive airway diseases	23.0	26.3	-0.08	23.3	23.9	-0.01	41.6	37.3	0.09	41.1	40.8	0.00	21.2	17.2	0.10	20.2	20.7	-0.01
Drugs used in diabetes	23.8	23.4	0.01	24.0	23.3	0.02	32.3	29.7	0.06	31.9	32.1	0.00	21.9	14.6	0.19	20.2	20.8	-0.01
Immunosuppressants	2.0	1.5	0.04	2.0	1.7	0.02	3.5	3.0	0.03	3.4	3.3	0.00	9.1	3.8	0.22	8.1	5.4	0.11
Lipid modifying agents	43.8	46.7	-0.06	44.1	43.1	0.02	68.3	68.0	0.01	68.3	68.1	0.00	49.6	41.7	0.16	47.9	54.6	-0.14
Opioids	11.9	12.4	-0.01	11.9	12.7	-0.02	15.2	11.6	0.11	14.7	14.6	0.00	22.7	14.2	0.22	20.4	20.9	-0.01
Psycholeptics	28.8	30.4	-0.04	29.0	29.5	-0.01	24.0	18.7	0.13	23.2	23.0	0.00	24.2	17.7	0.16	22.5	23.6	-0.03
Psychostimulants, ADHD agents and nootropics	3.3	3.9	-0.03	3.4	3.4	0.00	1.2	0.8	0.04	1.1	1.1	0.00	2.4	1.5	0.06	2.3	1.7	0.05
Race																		
American Indian or Alaska Native							0.6	0.6	0.01	0.6	0.6	0.00	<0.2	0.0		<0.2	0.0	
Asian							0.6	0.7	-0.01	0.6	0.6	0.00	2.2	1.5	-0.03	2.1	1.3	-0.01
Black or African American							16.1	12.2	0.11	15.5	15.4	0.00	8.3	5.1	0.13	7.6	9.3	-0.06
Native Hawaiian or Other Pacific Islander							0.7	0.7	0.00	0.7	0.7	0.00	1.1	<0.9	0.09	1.0	<0.9	0.08
White							76.1	79.7	-0.09	76.6	76.7	0.00	40.0	37.2	0.06	39.8	40.9	-0.02
Other or unknown							5.9	6.2	-0.01	6.0	6.0	0.00	2.0	2.7	-0.05	2.0	1.5	0.04
Ethnicity																		
Hispanic or Latino							5.2	5.4	-0.01	5.2	5.3	0.00	15.9	9.9	0.18	14.5	13.3	0.04
Not Hispanic nor Latino							92.3	91.9	0.01	92.2	92.2	0.00	38.9	36.5	0.05	38.6	37.8	0.02

Characteristic	OpenClaims						Optum DOD						Optum EHR					
	Before stratification N = 1,995,594 (T)			After stratification and 366,734 (C)			Before stratification N = 241,842 (T)			After stratification and 39,032 (C)			Before stratification N = 15,275 (T)			After stratification and 2,136 (C)		
	T (%)	C (%)	SDf	T (%)	C (%)	SDf	T (%)	C (%)	SDf	T (%)	C (%)	SDf	T (%)	C (%)	SDf	T (%)	C (%)	SDf
Age group																		
<25	0.1	0.1	0.00	0.1	0.1	0.00	0.0	0.0	0.00	0.0	0.0	0.00						
25-29	0.1	0.1	0.00	0.1	0.1	0.00	0.0	0.0	0.01	0.0	0.0	0.01	0.0	0.0		0.0	0.0	
30-34	0.1	0.1	-0.01	0.1	0.1	-0.01	0.0	0.0	0.00	0.0	0.0	0.00	0.0	0.0		0.0	0.0	
35-39	0.2	0.2	-0.01	0.2	0.2	0.00	0.1	0.1	0.01	0.1	0.1	0.00	0.1	<0.2	0.00	0.1	<0.2	0.00
40-44	0.4	0.4	0.01	0.4	0.4	0.01	0.3	0.2	0.03	0.3	0.2	0.03	0.6	0.4	0.02	0.5	0.3	0.04
45-49	1.2	0.8	0.04	1.2	1.0	0.01	0.8	0.5	0.04	0.8	0.6	0.02	1.6	0.7	0.08	1.5	0.7	0.07
50-54	3.1	1.8	0.08	2.9	2.7	0.01	2.0	1.1	0.08	1.9	1.7	0.01	4.0	2.2	0.10	3.9	2.6	0.07
55-59	7.0	4.1	0.13	6.6	6.2	0.02	4.9	2.6	0.12	4.5	4.4	0.01	9.0	5.3	0.14	8.6	8.0	0.02
60-64	12.2	8.1	0.14	11.6	11.3	0.01	8.5	5.3	0.12	8.0	8.0	0.00	15.3	10.2	0.16	14.6	14.8	-0.01
65-69	17.4	13.9	0.10	16.8	16.9	0.00	16.6	12.6	0.11	16.0	16.3	-0.01	17.8	14.3	0.10	17.4	17.7	-0.01
70-74	19.2	19.2	0.00	19.2	19.1	0.00	21.9	20.6	0.03	21.7	21.9	0.00	17.8	17.5	0.01	17.5	20.3	-0.07
75-79	16.7	19.2	-0.07	17.1	17.4	-0.01	19.7	21.9	-0.05	20.0	19.9	0.00	14.8	16.8	-0.05	15.1	15.1	0.00
80-84	17.1	24.1	-0.18	18.1	18.7	-0.02	13.3	17.2	-0.11	13.9	13.9	0.00	9.9	15.1	-0.16	10.7	10.3	0.01
85-89	5.3	7.8	-0.10	5.7	5.8	0.00	9.6	14.5	-0.15	10.3	10.4	0.00	9.0	17.4	-0.25	10.1	10.0	0.00
90-94							2.3	3.5	-0.07	2.4	2.5	-0.01						
95+																		
Gender: female																		
Medical history: General																		
Acute respiratory disease	7.1	5.8	0.05	6.9	6.9	0.00	13.2	11.3	0.06	12.9	13.1	-0.01	18.4	15.1	0.09	17.9	18.4	-0.01
Attention deficit hyperactivity disorder	0.3	0.2	0.02	0.3	0.3	0.00	0.5	0.3	0.04	0.5	0.4	0.01	0.7	0.7	0.01	0.7	0.9	-0.02
Chronic liver disease	0.7	0.4	0.05	0.7	0.6	0.01	1.3	0.8	0.05	1.3	1.2	0.00	2.9	1.6	0.09	2.8	2.6	0.01
Chronic obstructive lung disease	7.0	5.4	0.07	6.8	6.7	0.00	13.8	11.0	0.08	13.4	13.6	-0.01	17.7	13.9	0.10	17.2	17.3	0.00
Crohn's disease	0.2	0.2	0.01	0.2	0.2	0.00	0.3	0.3	0.01	0.3	0.3	0.00	0.5	0.4	0.02	0.5	0.5	0.00
Dementia	1.8	2.3	-0.03	1.9	1.9	0.00	5.2	6.3	-0.04	5.4	5.5	0.00	3.7	4.4	-0.03	3.8	3.3	0.03
Depressive disorder	4.6	3.4	0.06	4.5	4.3	0.01	11.6	9.0	0.09	11.3	11.2	0.00	15.2	11.1	0.12	14.6	15.3	-0.02
Diabetes mellitus	19.3	16.0	0.09	18.8	18.6	0.00	32.4	28.7	0.08	31.9	32.1	0.00	31.9	24.9	0.16	31.0	33.6	-0.06
Gastroesophageal reflux disease	6.7	5.6	0.05	6.6	6.4	0.01	17.2	15.2	0.06	16.9	17.1	-0.01	25.6	21.7	0.09	25.0	27.0	-0.04
Gastrointestinal hemorrhage	1.1	1.0	0.02	1.1	1.1	0.00	2.2	2.0	0.02	2.2	2.3	-0.01	3.3	2.6	0.04	3.3	3.1	0.01
Human immunodeficiency virus infection	0.3	0.3	0.01	0.3	0.3	0.00	0.4	0.4	0.01	0.4	0.5	-0.02	0.7	0.7	0.00	0.6	0.9	-0.03
Hyperlipidemia	29.8	28.9	0.02	29.7	29.6	0.00	60.8	61.2	-0.01	60.8	61.1	0.00	62.0	61.5	0.01	61.8	62.8	-0.02
Hypertensive disorder	38.2	34.8	0.07	37.7	37.6	0.00	66.9	64.5	0.05	66.6	66.8	0.00	66.2	63.5	0.06	65.8	67.1	-0.03
Lesion of liver	0.8	0.5	0.04	0.7	0.7	0.01	1.4	0.9	0.04	1.3	1.2	0.00	2.6	1.3	0.09	2.5	1.9	0.04
Obesity	3.9	2.7	0.07	3.7	3.5	0.01	11.9	8.7	0.10	11.5	11.4	0.00	19.4	12.6	0.19	18.5	20.7	-0.05
Osteoarthritis	13.5	11.3	0.07	13.2	12.9	0.01	27.0	23.7	0.08	26.6	26.7	0.00	26.9	22.2	0.11	26.3	27.8	-0.03
Pneumonia	2.0	1.7	0.02	2.0	1.9	0.00	3.9	3.3	0.03	3.8	4.0	-0.01	6.3	5.6	0.03	6.1	6.2	0.00
Psoriasis	0.6	0.5	0.01	0.6	0.6	0.00	1.2	1.0	0.01	1.2	1.2	0.00	1.4	1.2	0.01	1.3	1.4	0.00
Renal impairment	11.2	9.7	0.05	11.1	10.8	0.01	22.4	21.2	0.03	22.3	22.3	0.00	23.7	21.6	0.05	23.5	24.2	-0.02
Rheumatoid arthritis	0.5	0.4	0.01	0.5	0.5	0.00	1.1	0.9	0.02	1.1	1.1	0.00	1.4	1.0	0.04	1.4	1.4	0.00
Schizophrenia	0.3	0.1	0.03	0.2	0.2	0.01	0.5	0.3	0.04	0.4	0.4	0.01	0.5	<0.2	0.07	0.5	0.3	0.03
Ulcerative colitis	0.2	0.2	0.00	0.2	0.2	-0.01	0.4	0.4	-0.01	0.4	0.5	-0.01	0.6	0.5	0.01	0.6	0.6	-0.01
Urinary tract infectious disease	4.3	3.6	0.04	4.2	4.1	0.00	8.1	7.1	0.04	8.0	7.9	0.00	6.7	5.0	0.07	6.5	5.3	0.05
Viral hepatitis C	0.4	0.2	0.05	0.4	0.3	0.02	0.7	0.3	0.06	0.7	0.6	0.02	1.3	0.6	0.07	1.2	0.8	0.04
Visual system disorder	15.2	16.7	-0.04	15.4	15.8	-0.01	34.5	38.1	-0.07	35.0	34.9	0.00	16.0	15.7	0.01	15.9	17.0	-0.03

(Continued)

Characteristic	OpenClaims						Optum DOD						Optum EHR					
	Before stratification $N = 1{,}995{,}594$ (T)			After stratification and 366,734 (C)			Before stratification $N = 241{,}842$ (T)			After stratification and 39,032 (C)			Before stratification $N = 15{,}275$ (T)			After stratification and 2,136 (C)		
	T (%)	C (%)	SDf	T (%)	C (%)	SDf	T (%)	C (%)	SDf	T (%)	C (%)	SDf	T (%)	C (%)	SDf	T (%)	C (%)	SDf
Medical history: Cardiovascular disease																		
Atrial fibrillation	7.5	8.6	-0.04	7.7	7.8	0.00	14.0	16.0	-0.05	14.3	14.4	0.00	16.4	20.7	-0.11	16.9	16.8	0.00
Cerebrovascular disease	3.3	3.2	0.00	3.3	3.3	0.00	7.4	7.3	0.00	7.4	7.4	0.00	7.6	7.4	0.01	7.6	8.2	-0.02
Coronary arteriosclerosis	11.5	11.3	0.01	11.5	11.7	-0.01	23.6	23.4	0.00	23.5	23.7	0.00	27.9	26.1	0.04	27.8	27.8	0.00
Heart disease	23.5	23.7	0.00	23.5	23.8	-0.01	41.8	42.5	-0.01	41.9	42.1	0.00	47.8	47.9	0.00	47.8	48.3	-0.01
Heart failure	5.7	5.3	0.01	5.6	5.6	0.00	11.4	10.8	0.02	11.4	11.5	0.00	14.0	13.0	0.03	13.9	14.2	-0.01
Ischemic heart disease	4.0	3.6	0.03	4.0	4.0	0.00	10.0	9.2	0.02	9.9	9.9	0.00	14.5	12.7	0.05	14.3	15.4	-0.03
Peripheral vascular disease	4.0	3.5	0.03	3.9	3.9	0.00	11.4	10.2	0.04	11.2	11.3	0.00	10.6	9.8	0.03	10.4	12.7	-0.07
Pulmonary embolism	0.5	0.5	0.00	0.5	0.5	0.00	0.9	1.0	-0.01	0.9	1.0	-0.01	1.5	1.1	0.04	1.5	1.3	0.01
Venous thrombosis	0.9	0.9	0.00	0.9	0.9	0.00	1.8	1.9	-0.01	1.9	1.9	0.00	2.3	2.5	-0.01	2.3	3.2	-0.06
Medical history: Neoplasms																		
Hematologic neoplasm	1.2	1.2	0.00	1.2	1.2	0.00	2.0	2.0	0.00	2.0	1.9	0.00	3.3	3.1	0.01	3.2	3.3	0.00
Malignant lymphoma	0.7	0.8	-0.01	0.8	0.8	0.00	1.1	1.2	-0.01	1.1	1.1	0.00	1.5	1.7	-0.01	1.6	1.6	0.00
Malignant neoplasm of anorectum	0.2	0.1	0.02	0.2	0.2	0.01	0.3	0.2	0.02	0.3	0.2	0.01	0.5	0.2	0.04	0.5	0.3	0.03
Malignant neoplastic disease	11.1	9.7	0.04	10.9	10.7	0.01	17.8	16.9	0.03	17.7	17.3	0.01	19.7	18.4	0.03	19.5	19.9	-0.01
Malignant tumor of breast	0.0	0.0	0.00	0.0	0.0	0.00	0.1	0.0	0.01	0.1	0.0	0.01	0.1	<0.2	0.00	0.1	<0.2	0.01
Malignant tumor of colon	0.4	0.3	0.01	0.4	0.4	0.00	0.7	0.6	0.02	0.7	0.6	0.01	0.8	0.7	0.01	0.8	0.5	0.04
Malignant tumor of lung	0.5	0.4	0.01	0.5	0.5	0.00	0.8	0.6	0.02	0.8	0.7	0.01	1.4	0.9	0.04	1.3	1.2	0.01
Malignant tumor of urinary bladder	0.9	0.9	0.00	0.9	0.9	0.00	1.3	1.7	-0.03	1.4	1.5	-0.01	2.0	2.6	-0.04	2.1	2.8	-0.05
Primary malignant neoplasm of prostate	4.8	2.9	0.10	4.6	4.2	0.02	7.3	4.8	0.11	7.0	6.7	0.01	5.8	3.0	0.14	5.5	4.7	0.04
Medication use																		
Antibacterials for systemic use	43.9	40.5	0.07	43.4	43.3	0.00	42.1	38.4	0.08	41.6	41.6	0.00	49.4	42.3	0.14	48.5	48.8	-0.01
Antidepressants	24.0	19.6	0.11	23.3	23.4	0.00	24.3	20.4	0.09	23.8	24.1	-0.01	32.1	26.4	0.13	31.3	33.1	-0.04
Antiepileptics	18.9	14.3	0.12	18.2	18.0	0.01	19.8	15.3	0.12	19.2	19.4	-0.01	26.8	19.3	0.18	25.7	28.2	-0.06
Antiinflammatory and antirheumatic products	26.0	19.6	0.15	25.0	24.6	0.01	20.7	16.2	0.11	20.1	19.7	0.01	33.0	25.1	0.17	31.8	34.3	-0.05
Antineoplastic agents	5.5	5.4	0.00	5.5	5.6	0.00	4.0	3.9	0.01	4.0	4.0	0.00	5.2	5.3	-0.01	5.1	5.3	-0.01
Antipsoriatics	1.0	0.9	0.01	1.0	1.0	0.00	1.0	0.8	0.02	1.0	0.9	0.00	1.6	1.0	0.06	1.6	1.2	0.03
Antithrombotic agents	25.5	24.8	0.01	25.4	25.5	0.00	24.7	25.1	-0.01	24.8	25.1	-0.01	61.2	59.7	0.03	60.9	62.6	-0.04
Drugs for acid related disorders	32.0	27.6	0.10	31.3	31.4	0.00	31.2	28.2	0.06	30.8	31.0	0.00	52.2	46.1	0.12	51.4	53.6	-0.04
Drugs for obstructive airway diseases	31.7	27.7	0.09	31.1	31.0	0.00	23.6	19.6	0.10	23.0	23.3	-0.01	44.7	36.6	0.17	43.6	44.0	-0.01
Drugs used in diabetes	26.1	21.5	0.11	25.5	25.4	0.00	28.0	24.2	0.09	27.5	27.8	-0.01	32.5	24.7	0.17	31.5	34.3	-0.06
Immunosuppressants	2.8	2.3	0.03	2.8	2.7	0.00	2.8	2.3	0.03	2.7	2.7	0.00	4.8	3.5	0.07	4.7	4.1	0.03
Lipid modifying agents	61.5	61.9	-0.01	61.6	62.2	-0.01	65.3	66.7	-0.03	65.5	65.8	-0.01	67.0	66.7	0.00	66.9	69.1	-0.05
Opioids	18.8	13.6	0.14	18.0	17.4	0.02	19.0	14.0	0.13	18.3	18.0	0.01	32.6	24.8	0.17	31.3	34.1	-0.06
Psycholeptics	19.2	16.2	0.08	18.8	18.7	0.00	20.2	16.9	0.08	19.7	19.7	0.00	38.4	30.2	0.17	37.2	38.6	-0.03
Psychostimulants, ADHD agents and nootropics	1.8	1.4	0.03	1.7	1.8	0.00	1.3	0.8	0.05	1.2	1.2	0.00	3.0	2.2	0.06	2.9	3.3	-0.02
Race																		
American Indian or Alaska Native																		
Asian													1.3	1.6	-0.02	1.3	1.5	-0.02
Black or African American													8.5	5.5	0.12	8.2	7.1	0.04
Native Hawaiian or Other Pacific Islander																		
White													83.8	88.0	-0.12	84.3	85.1	-0.02
Other or unknown																		
Ethnicity																		
Hispanic or Latino													3.9	3.2	0.04	3.8	4.1	-0.01
Not Hispanic nor Latino													86.5	87.9	-0.04	86.6	86.3	0.01

Negative control calibration: estimates before and after

Negative control experiment uses a large number of “true negatives,” outcomes to which the exposures have no known relation, to detect potential biases induced by residual confoundings. The experiment also diagnoses potential under- or over-coverage of the null effect by the estimated CIs. These results can in turn be used to calibrate our estimates and confidence intervals for the actual clinical questions of interest to correct for potential biases and miscalibrated uncertainty estimates. In practice, negative control calibration typically results in more conservative conclusions with fewer statistically significant findings.

To demonstrate the process of negative control calibration, we first consider what our analysis would have produced had we not adequately addressed confounding. Supplementary Figure 2(a) below shows the results of negative control experiments in comparing the alpha-1 blocker and 5ARI/PDE5 cohorts if no covariates were to be adjusted. Residual confounding is evident. The uncalibrated estimates show that the alpha-1 blocker users tend to have higher hazard ratios on average across the variety of health outcomes. This could indicate, for example, that alpha-1 blocker users have worse baseline health. The calibration debiases the original estimates, in this case by lowering hazard ratios; graphically, the process manifests itself as the calibrated blue dots being shifted leftward compared to the uncalibrated orange ones. The calibration also widens the confidence intervals, with the blue dots shifted upward compared to the orange dots.

The presence of substantial confounding is no surprise in the toy example above. Even when we control for measured confounders, however, there remains substantial concern for residual confounding. This is where negative control experiments become essential. Supplementary Figure 2(b) shows the results of negative control experiments in comparing the alpha-1 blocker and 5ARI/PDE5 cohorts using our large-scale PS stratification analysis. The negative control experiments here reveal at most minor residual confoundings, with the estimates and CIs demonstrating resonable performance without calibration. Correspondingly, the calibration has rather small effects on the estimates. We also provide numeric values of the uncalibrated estimates in Supplementary Table 3, following the format of Table 3 in the main text.

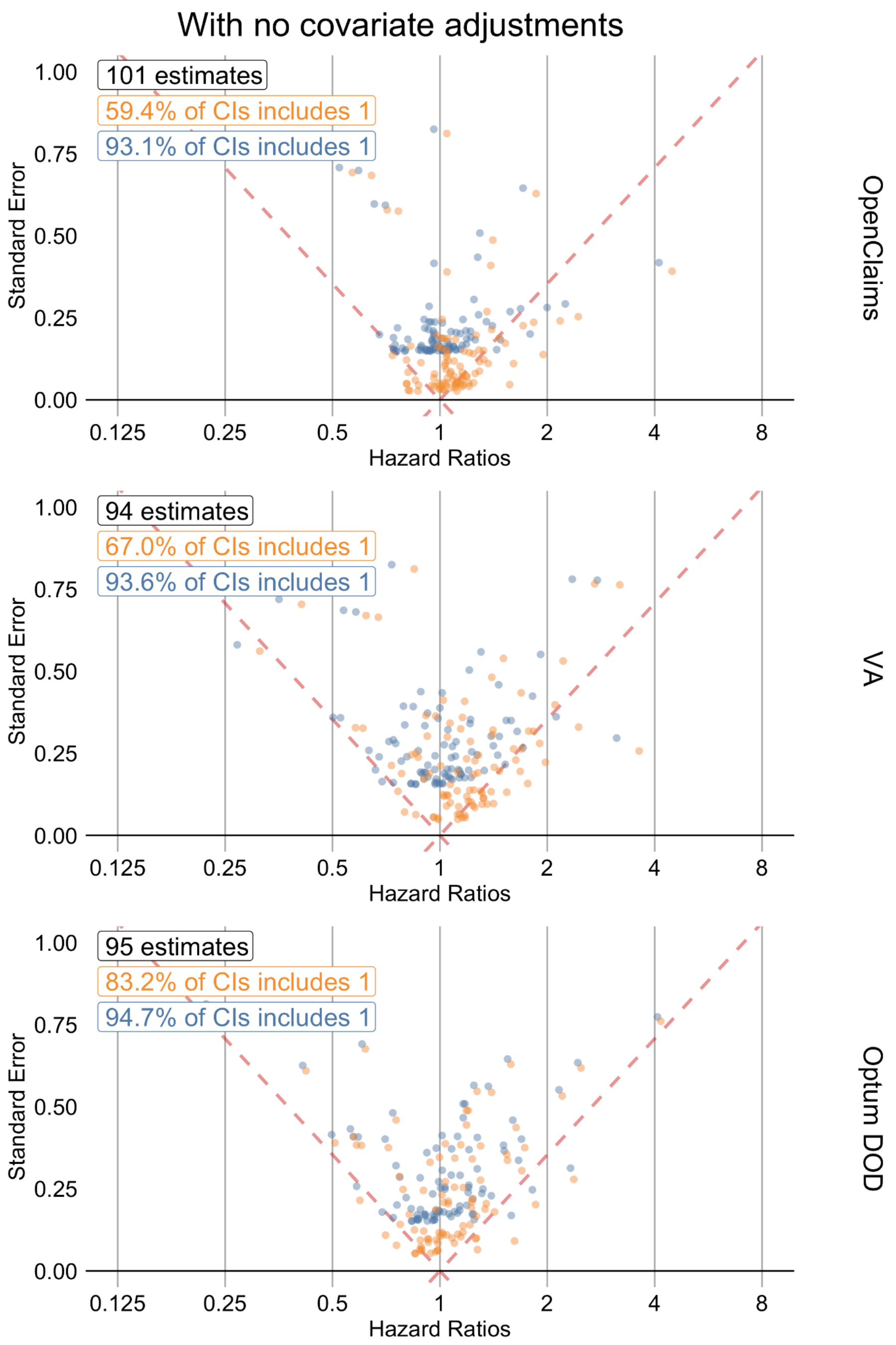

Supplementary Figure 2(a): Estimated hazard ratios and standard errors for negative control outcomes when no covariates are adjusted. Orange dots represent original uncalibrated estimates, while blue dots represent calibrated estimates. Shown here are the results for the three larger databases in terms of study cohort sizes (OpenClaims, VA, and Optum DOD).

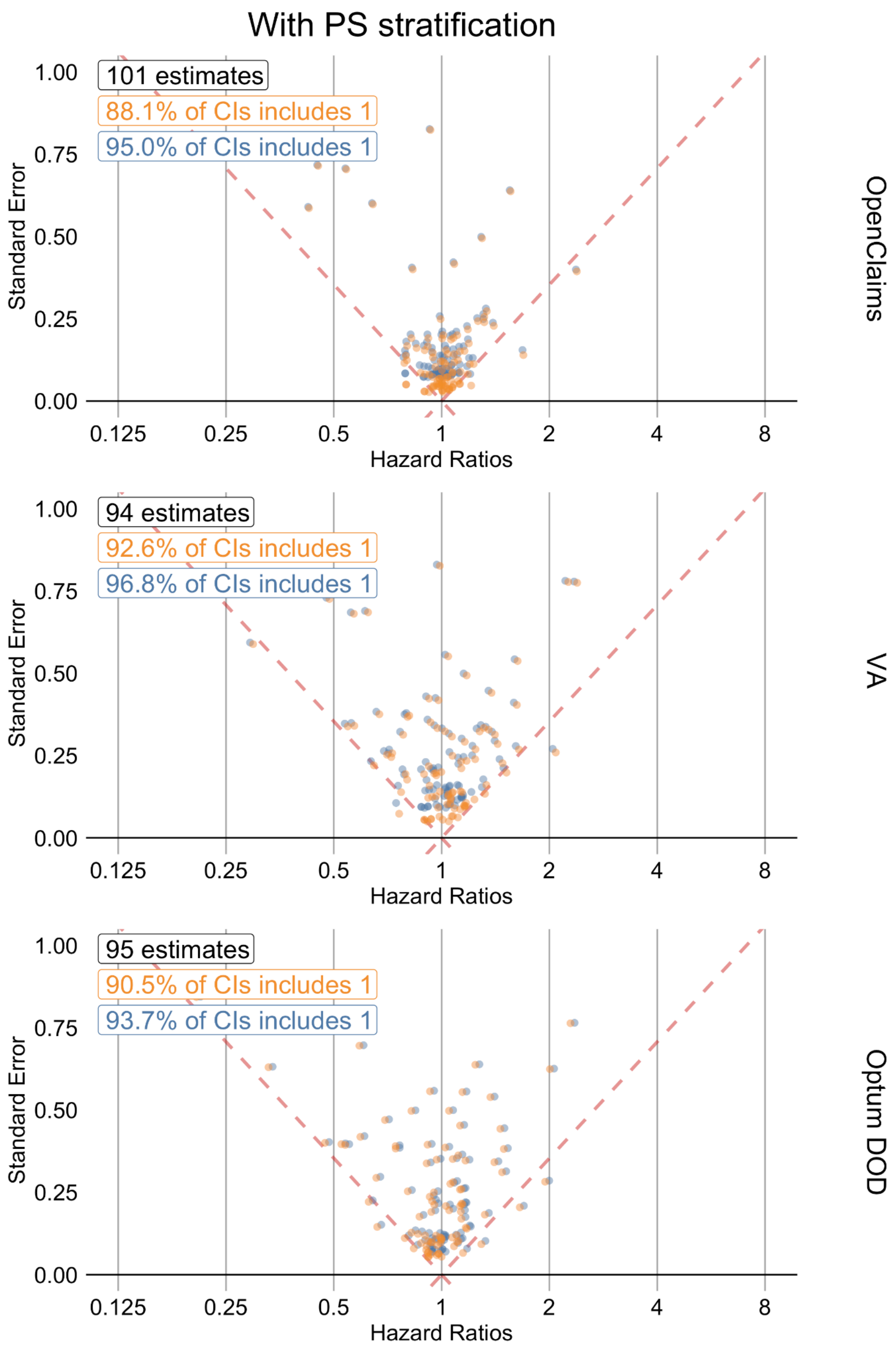

Supplementary Figure 2(b): Estimated hazard ratios and standard errors for negative control outcomes when PS stratification is deployed. Compared to the unadjusted case in Figure 1(a), the actual null coverage by the CIs is close to the nominal 95% value even before calibration. This suggests that the stratification by our large-scale PS model has successfully removed most of confoundings.

Supplementary Table 2: Uncalibrated hazard ratios, 95% CIs, and p-values for the three COVID-19 outcomes under the stratified and matched analysis across all the six databases. The estimates and CIs are comparable to those of calibrated estiamtes shown in Table 3 of the main text and, if any, tend to be more conservative overall.

	PS-stratified			PS-matched		
	HR	95% CI	p	HR	95% CI	p
Diagnosis						
SIDIAP	0.90	(0.67 - 1.23)	0.50	0.83	(0.61 - 1.16)	0.26
VA	1.04	(0.91 - 1.20)	0.55	1.05	(0.91 - 1.22)	0.52
CUIMC	0.96	(0.34 - 3.42)	0.94	1.66	(0.39 - 11.4)	0.56
OpenClaims	1.05	(0.97 - 1.14)	0.21	1.05	(0.97 - 1.14)	0.25
Optum DOD	0.67	(0.49 - 0.94)	0.02	0.73	(0.50 - 1.08)	0.10
Optum EHR	1.00	(0.47 - 2.46)	1.00	1.32	(0.39 - 5.11)	0.67
Meta-analysis	1.03	(0.96 - 1.10)	0.46	1.01	(0.93 - 1.11)	0.76
+ Hospitalization						
SIDIAP	1.00	(0.64 - 1.67)	0.99	0.88	(0.53 - 1.52)	0.63
VA	0.91	(0.73 - 1.14)	0.40	0.90	(0.72 - 1.16)	0.41
CUIMC	2.38	(0.43 - 44.8)	0.46	2.70	(0.40 - 53.1)	0.43
OpenClaims	1.09	(0.98 - 1.21)	0.13	1.06	(0.95 - 1.18)	0.30
Optum DOD	0.62	(0.43 - 0.92)	0.01	0.75	(0.48 - 1.18)	0.20
Optum EHR	0.83	(0.34 - 2.47)	0.71	1.00	(0.27 - 4.08)	1.00
Meta-analysis	0.98	(0.86 - 1.13)	0.80	0.99	(0.89 - 1.11)	0.91
+ Intensive services						
SIDIAP	NA	NA	NA	NA	NA	NA
VA	1.26	(0.71 - 2.44)	0.46	1.27	(0.70 - 2.51)	0.46
CUIMC	NA	NA	NA	NA	NA	NA
OpenClaims	NA	NA	NA	NA	NA	NA
Optum DOD	0.54	(0.22 - 1.52)	0.21	0.68	(0.21 - 2.63)	0.55
Optum EHR	NA	NA	NA	NA	NA	NA
Meta-analysis	1.15	(0.74 - 1.79)	0.52	1.15	(0.71 - 1.86)	0.58

Cohort and exposure definitions in OMOP Common Data Model

Exposures

1. Alpha-blockers

Concept Id	Concept Name	Domain	Vocabulary	Excluded	Descendants	Mapped
924566	tamsulosin	Drug	RxNorm	NO	YES	NO
930021	alfuzosin	Drug	RxNorm	NO	YES	NO
1341238	terazosin	Drug	RxNorm	NO	YES	NO
1350489	prazosin	Drug	RxNorm	NO	YES	NO

1363053	doxazosin	Drug	RxNorm	NO	YES	NO
19012925	silodosin	Drug	RxNorm	NO	YES	NO

2. 5-alpha reductase inhibitors / phosphodiesterase type 5 inhibitor (indicated for BPH)

Concept Id	Concept Name	Domain	Vocabulary	Excluded	Descendants	Mapped
989482	dutasteride	Drug	RxNorm	NO	YES	NO
996416	finasteride	Drug	RxNorm	NO	YES	NO
1336926	tadalafil	Drug	RxNorm	NO	YES	NO

3. BPH

Concept Id	Concept Name	Domain	Vocabulary	Excluded	Descendants	Mapped
198803	Benign prostatic hyperplasia	Condition	SNOMED	NO	YES	NO

Outcome Cohort

This section documents the outcome cohort definitions. We consider

- COVID-19 diagnosis or SARS-CoV-2 positive test with no required prior observation
- Hospitalization with a COVID-19 diagnosis record or SARS-CoV-2 positive test with no required prior observation
- Hospitalization and requiring intensive services with a SARS-CoV-2 positive test with no required prior observation

Below are their complete specifications.

[COVID ID133 V1] Persons with a COVID-19 diagnosis or a SARS-CoV-2 positive test with no required prior observation

Initial Event Cohort

People having any of the following:

- a measurement of SARS-CoV-2 positive test measurement pre-coordinated[2]
    - occurrence start is after 2019-12-01
- a measurement of SARS-CoV-2 test measurement[3]
    - occurrence start is after 2019-12-01
    - value as concept is any of: Detected, Detected, Positive, Positive, Present, Present
- an observation of SARS-CoV-2 test measurement[3]
    - occurrence start is after 2019-12-01
    - value as concept is any of: Detected, Detected, Positive, Positive, Present, Present
- a condition occurrence of COVID-19 conditions[1]
    - occurrence start is after 2019-12-01

with continuous observation of at least 0 days prior and 0 days after event index date, and limit initial events to: **earliest event per person.**

Limit qualifying cohort to: **earliest event per person.**

End Date Strategy

Date Offset Exit Criteria
This cohort defintion end date will be the index event's start date plus 1 days

Cohort Collapse Strategy:
Collapse cohort by era with a gap size of 90 days.

1. COVID-19 conditions

Concept Id	Concept Name	Domain	Vocabulary	Excluded	Descendants	Mapped
439676	Coronavirus infection	Condition	SNOMED	NO	YES	NO
4100065	Disease due to Coronaviridae	Condition	SNOMED	NO	YES	NO
37311060	Suspected disease caused by 2019-nCoV	Observation	SNOMED	NO	YES	NO
37311061	Disease caused by 2019-nCoV	Condition	SNOMED	NO	YES	NO

2. SARS-CoV-2 positive test measurement pre-coordinated

Concept Id	Concept Name	Domain	Vocabulary	Excluded	Descendants	Mapped
37310282	2019 novel coronavirus detected	Measurement	SNOMED	NO	YES	NO

3. SARS-CoV-2 test measurement

Concept Id	Concept Name	Domain	Vocabulary	Excluded	Descendants	Mapped
756055	Measurement of Severe acute respiratory syndrome coronavirus 2 (SARS-CoV-2)	Measurement	OMOP Extension	NO	YES	NO
37310281	2019 novel coronavirus not detected	Measurement	SNOMED	YES	YES	NO

[COVID ID135 V1] Persons hospitalized with a COVID-19 diagnosis record or a SARS-CoV-2 positive test with no required prior observation

Initial Event Cohort

People having any of the following:

- a visit occurrence of Inpatient Visit[2]
    - occurrence start is after 2019-12-01

with continuous observation of at least 0 days prior and 0 days after event index date, and limit initial events to: **all events per person.**

For people matching the Primary Events, include:
Having any of the following criteria:

- at least 1 occurrences of a measurement of SARS-CoV-2 positive test measurement pre-coordinated[3]
- where event starts between 21 days Before and all days After index start date and event starts between all days Before and 0 days After index end date
- or at least 1 occurrences of a measurement of SARS-CoV-2 test measurement[4]
    - value as concept is any of: Detected, Detected, Positive, Positive, Present, Present
- where event starts between 21 days Before and all days After index start date and event starts between all days Before and 0 days After index end date
- or at least 1 occurrences of an observation of SARS-CoV-2 test measurement[4]
    - value as concept is any of: Detected, Detected, Positive, Positive, Present, Present
- where event starts between 21 days Before and all days After index start date and event starts between all days Before and 0 days After index end date
- or at least 1 occurrences of a condition occurrence of COVID-19 conditions[1]
- where event starts between 21 days Before and all days After index start date and event starts between all days Before and 0 days After index end date

Limit cohort of initial events to: **earliest event per person.**
Limit qualifying cohort to: **earliest event per person.**

End Date Strategy

Date Offset Exit Criteria
This cohort defintion end date will be the index event's end date plus 0 days

Cohort Collapse Strategy:
Collapse cohort by era with a gap size of 90 days.

1. COVID-19 conditions

Concept Id	Concept Name	Domain	Vocabulary	Excluded	Descendants	Mapped
439676	Coronavirus infection	Condition	SNOMED	NO	YES	NO
4100065	Disease due to Coronaviridae	Condition	SNOMED	NO	YES	NO
37311060	Suspected disease caused by 2019-nCoV	Observation	SNOMED	NO	YES	NO
37311061	Disease caused by 2019-nCoV	Condition	SNOMED	NO	YES	NO

2. Inpatient Visit

Concept Id	Concept Name	Domain	Vocabulary	Excluded	Descendants	Mapped
262	Emergency Room and Inpatient Visit	Visit	Visit	NO	YES	NO
9201	Inpatient Visit	Visit	Visit	NO	YES	NO

3. SARS-CoV-2 positive test measurement pre-coordinated

Concept Id	Concept Name	Domain	Vocabulary	Excluded	Descendants	Mapped
37310282	2019 novel coronavirus detected	Measurement	SNOMED	NO	YES	NO

4. SARS-CoV-2 test measurement

Concept Id	Concept Name	Domain	Vocabulary	Excluded	Descendants	Mapped
756055	Measurement of Severe acute respiratory syndrome coronavirus 2 (SARS-CoV-2)	Measurement	OMOP Extension	NO	YES	NO
37310281	2019 novel coronavirus not detected	Measurement	SNOMED	YES	YES	NO

[COVID ID137 V1] Persons hospitalized and requiring intensive services with a COVID-19 diagnosis record or a SARS-CoV-2 positive test with no required prior observation

Initial Event Cohort

People having any of the following:

- a procedure of Mechanical ventilation[4]
  - occurrence start is after 2019-12-01
- a condition occurrence of Mechanical ventilation[4]
  - occurrence start is after 2019-12-01
- an observation of Mechanical ventilation[4]
  - occurrence start is after 2019-12-01
- a procedure of tracheostomy[7]
  - occurrence start is after 2019-12-01
- a procedure of Extracorporeal membrane oxygenation (ECMO) procedure[2]
  - occurrence start is after 2019-12-01

with continuous observation of at least 0 days prior and 0 days after event index date, and limit initial events to: **all events per person.**

For people matching the Primary Events, include:
Having all of the following criteria:

- at least 1 occurrences of a visit occurrence of Inpatient Visit[3]

- Having any of the following criteria:
  - at least 1 occurrences of a measurement of SARS-CoV-2 positive test measurement pre-coordinated[5]
  - where event starts between 21 days Before and all days After index start date and event starts between all days Before and 0 days After index end date
  - or at least 1 occurrences of a measurement of SARS-CoV-2 test measurement[6]
    - value as concept is any of: Detected, Detected, Positive, Positive, Present, Present
  - where event starts between 21 days Before and all days After index start date and event starts between all days Before and 0 days After index end date
  - or at least 1 occurrences of an observation of SARS-CoV-2 test measurement[6]
    - value as concept is any of: Detected, Detected, Positive, Positive, Present, Present
  - where event starts between 21 days Before and all days After index start date and event starts between all days Before and 0 days After index end date
  - or at least 1 occurrences of a condition occurrence of COVID-19 conditions[1]
  - where event starts between 21 days Before and all days After index start date and event starts between all days Before and 0 days After index end date
- where event starts between all days Before and 0 days After index start date and event ends between 0 days Before and all days After index start date

Limit cohort of initial events to: **earliest event per person.**
Limit qualifying cohort to: **earliest event per person.**

End Date Strategy

Date Offset Exit Criteria
This cohort defintion end date will be the index event's end date plus 0 days

Cohort Collapse Strategy:
Collapse cohort by era with a gap size of 90 days.

1. COVID-19 conditions

Concept Id	Concept Name	Domain	Vocabulary	Excluded	Descendants	Mapped
439676	Coronavirus infection	Condition	SNOMED	NO	YES	NO
4100065	Disease due to Coronaviridae	Condition	SNOMED	NO	YES	NO
37311060	Suspected disease caused by 2019-nCoV	Observation	SNOMED	NO	YES	NO
37311061	Disease caused by 2019-nCoV	Condition	SNOMED	NO	YES	NO

2. Extracorporeal membrane oxygenation (ECMO) procedure

Concept Id	Concept Name	Domain	Vocabulary	Excluded	Descendants	Mapped

1531630	Extracorporeal Oxygenation, Membrane, Peripheral Veno-venous	Procedure	ICD10PCS	NO	NO	NO
1531631	Extracorporeal Oxygenation, Membrane, Peripheral Veno-arterial	Procedure	ICD10PCS	NO	NO	NO
1531632	Extracorporeal Oxygenation, Membrane, Central	Procedure	ICD10PCS	NO	NO	NO
2002247	Extracorporeal membrane oxygenation [ECMO]	Procedure	ICD9Proc	NO	YES	NO
2787820	Extracorporeal Supersaturated Oxygenation, Intermittent	Procedure	ICD10PCS	NO	NO	NO
2787821	Extracorporeal Hyperbaric Oxygenation, Continuous	Procedure	ICD10PCS	NO	NO	NO
4052536	Extracorporeal membrane oxygenation	Procedure	SNOMED	NO	YES	NO
4338595	Cardiac support using extracorporeal membrane oxygenation circuitry	Procedure	SNOMED	NO	NO	NO
44515635	Extracorporeal membrane oxygenation	Procedure	OPCS4	NO	YES	NO
44811012	Fluoroscopy guided percutaneous insertion of cannula for extracorporeal membrane oxygenation	Procedure	SNOMED	NO	NO	NO

3. Inpatient Visit

Concept Id	Concept Name	Domain	Vocabulary	Excluded	Descendants	Mapped
262	Emergency Room and Inpatient Visit	Visit	Visit	NO	YES	NO
9201	Inpatient Visit	Visit	Visit	NO	YES	NO

4. Mechanical ventilation

Concept Id	Concept Name	Domain	Vocabulary	Excluded	Descendants	Mapped
765576	Orotracheal intubation using bougie device	Procedure	SNOMED	NO	YES	NO
2108641	Glossectomy; complete or total, with or without tracheostomy, without radical neck dissection	Procedure	CPT4	YES	YES	NO
2108642	Glossectomy; complete or total, with or without tracheostomy, with unilateral radical neck dissection	Procedure	CPT4	YES	YES	NO
2108681	Patient receiving care in the intensive care unit (ICU) and receiving mechanical ventilation, 24 hours or less (CRIT)	Observation	CPT4	NO	YES	NO
2788036	Respiratory Ventilation, Less than 24 Consecutive Hours	Procedure	ICD10PCS	NO	YES	NO
2788037	Respiratory Ventilation, 24-96 Consecutive Hours	Procedure	ICD10PCS	NO	YES	NO
2788038	Respiratory Ventilation, Greater than 96 Consecutive Hours	Procedure	ICD10PCS	NO	YES	NO
4006318	Respiratory assist, manual	Procedure	SNOMED	YES	YES	NO
4021786	Fear of disconnection from ventilator	Condition	SNOMED	YES	YES	NO
4031379	Artificial ventilation finding	Condition	SNOMED	YES	YES	NO
4072633	Weaning from mechanically assisted ventilation	Procedure	SNOMED	NO	YES	NO
4074663	Diaphragmatic augmentation	Procedure	SNOMED	YES	YES	NO
4080957	Endotracheal respiratory assistance	Procedure	SNOMED	NO	YES	NO

4107247	Inhalation anesthesia, machine system, semi-closed, no rebreathing of primary agent	Procedure	SNOMED	YES	YES	NO
4168966	Endotracheal tube present	Observation	SNOMED	NO	YES	NO
4219858	Problem with patient ventilator	Observation	SNOMED	NO	YES	NO
4230167	Artificial respiration	Procedure	SNOMED	NO	YES	NO
4232550	Home visit for mechanical ventilation care	Observation	SNOMED	NO	YES	NO
4232891	Mechanical ventilation response	Observation	SNOMED	YES	YES	NO
4235361	Hyperventilation therapy for traumatic brain injury	Procedure	SNOMED	NO	YES	NO
4237618	Ventilator care	Observation	SNOMED	NO	YES	NO
4251737	Ventilator care management	Procedure	SNOMED	NO	YES	NO
4254108	Resuscitation with artificial respiration	Procedure	SNOMED	YES	YES	NO
4254905	Ventilator care education	Procedure	SNOMED	YES	YES	NO
4259233	Ventilator care assessment	Procedure	SNOMED	YES	YES	NO
4332501	Management of noninvasive mechanical ventilation	Procedure	SNOMED	NO	YES	NO
4348300	Expired air ventilation	Procedure	SNOMED	YES	YES	NO
4353715	Ventilator finding	Observation	SNOMED	YES	YES	NO
37116689	Insertion of endotracheal ventilation catheter	Procedure	SNOMED	NO	YES	NO
37206832	Mechanical insufflation exsufflation	Procedure	SNOMED	NO	YES	NO
40481547	Dependence on ventilator	Condition	SNOMED	NO	YES	NO
40487536	Intubation of respiratory tract	Procedure	SNOMED	NO	YES	NO

42738852	Ventilation assist and management, initiation of pressure or volume preset ventilators for assisted or controlled breathing; first day	Procedure	CPT4	NO	YES	NO
42738853	Ventilation assist and management, initiation of pressure or volume preset ventilators for assisted or controlled breathing; subsequent days	Procedure	CPT4	NO	YES	NO
44509482	Other specified ventilation support	Procedure	OPCS4	NO	YES	NO
44791135	Ventilatory support	Procedure	SNOMED	NO	YES	NO
44808555	Provision of mechanical ventilator	Procedure	SNOMED	YES	YES	NO
45887795	Ventilation assist and management, initiation of pressure or volume preset ventilators for assisted or controlled breathing	Procedure	CPT4	NO	YES	NO

Concept Id	Concept Name	Domain	Vocabulary	Excluded	Descendants	Mapped
765576	Orotracheal intubation using bougie device	Procedure	SNOMED	NO	YES	NO
2108641	Glossectomy; complete or total, with or without tracheostomy, without radical neck dissection	Procedure	CPT4	YES	YES	NO
2108642	Glossectomy; complete or total, with or without tracheostomy, with unilateral radical neck dissection	Procedure	CPT4	YES	YES	NO
2108681	Patient receiving care in the intensive care unit (ICU) and receiving mechanical ventilation, 24 hours or less (CRIT)	Observation	CPT4	NO	YES	NO

2788036	Respiratory Ventilation, Less than 24 Consecutive Hours	Procedure	ICD10PCS	NO	YES	NO
2788037	Respiratory Ventilation, 24-96 Consecutive Hours	Procedure	ICD10PCS	NO	YES	NO
2788038	Respiratory Ventilation, Greater than 96 Consecutive Hours	Procedure	ICD10PCS	NO	YES	NO
4006318	Respiratory assist, manual	Procedure	SNOMED	YES	YES	NO
4021786	Fear of disconnection from ventilator	Condition	SNOMED	YES	YES	NO
4031379	Artificial ventilation finding	Condition	SNOMED	YES	YES	NO

5. SARS-CoV-2 positive test measurement pre-coordinated

Concept Id	Concept Name	Domain	Vocabulary	Excluded	Descendants	Mapped
37310282	2019 novel coronavirus detected	Measurement	SNOMED	NO	YES	NO

6. SARS-CoV-2 test measurement

Concept Id	Concept Name	Domain	Vocabulary	Excluded	Descendants	Mapped
756055	Measurement of Severe acute respiratory syndrome coronavirus 2 (SARS-CoV-2)	Measurement	OMOP Extension	NO	YES	NO
37310281	2019 novel coronavirus not detected	Measurement	SNOMED	YES	YES	NO

7. tracheostomy

Concept Id	Concept Name	Domain	Vocabulary	Excluded	Descendants	Mapped
45887989	Transoral approach to skull base, brain stem or upper spinal cord for biopsy, decompression or excision of lesion	Procedure	CPT4	NO	YES	NO
4337047	Insertion of tracheostomy tube	Procedure	SNOMED	NO	YES	NO

4311023	Revision of stoma of trachea	Procedure	SNOMED	NO	YES	NO
4208093	Tracheostomy, emergency procedure by transtracheal approach	Procedure	SNOMED	NO	YES	NO
4199580	Mediastinal tracheostomy	Procedure	SNOMED	NO	YES	NO
4195473	Temporary tracheostomy	Procedure	SNOMED	NO	YES	NO
4168864	Lateral tracheostomy	Procedure	SNOMED	NO	YES	NO
4166281	Anterior tracheostomy	Procedure	SNOMED	NO	YES	NO
4115488	Emergency tracheostomy	Procedure	SNOMED	NO	YES	NO
4065590	Permanent tracheostomy	Procedure	SNOMED	NO	YES	NO
2870619	Medical and Surgical @ Respiratory System @ Revision @ Trachea @ Percutaneous @ Tracheostomy Device	Procedure	ICD10PCS	NO	YES	NO
2862930	Medical and Surgical @ Respiratory System @ Revision @ Trachea @ Open @ Tracheostomy Device	Procedure	ICD10PCS	NO	YES	NO
2836115	Medical and Surgical @ Respiratory System @ Bypass @ Trachea @ Percutaneous @ Tracheostomy Device	Procedure	ICD10PCS	NO	YES	NO
2831237	Medical and Surgical @ Respiratory System @ Bypass @ Trachea @ Open @ Tracheostomy Device	Procedure	ICD10PCS	NO	YES	NO
2829386	Medical and Surgical @ Respiratory System @ Revision @ Trachea @ Via Natural or Artificial	Procedure	ICD10PCS	NO	YES	NO

	Opening @ Tracheostomy Device					
2829384	Medical and Surgical @ Respiratory System @ Revision @ Trachea @ Percutaneous Endoscopic @ Tracheostomy Device	Procedure	ICD10PCS	NO	YES	NO
2794811	Medical and Surgical @ Respiratory System @ Change @ Trachea @ External @ Tracheostomy Device	Procedure	ICD10PCS	NO	YES	NO
2743216	Removal of Tracheostomy Device from Trachea, Via Natural or Artificial Opening	Procedure	ICD10PCS	NO	YES	NO
2110486	Transoral approach to skull base, brain stem or upper spinal cord for biopsy, decompression or excision of lesion; requiring splitting of tongue and/or mandible (including tracheostomy)	Procedure	CPT4	YES	YES	NO
2106569	Tracheal puncture, percutaneous with transtracheal aspiration and/or injection	Procedure	CPT4	NO	YES	NO
2106568	Construction of tracheoesophageal fistula and subsequent insertion of an alaryngeal speech prosthesis (eg, voice button, Blom-Singer prosthesis)	Procedure	CPT4	NO	YES	NO
2106567	Tracheostomy, fenestration procedure with skin flaps	Procedure	CPT4	NO	YES	NO

2106565	Tracheostomy, emergency procedure; cricothyroid membrane	Procedure	CPT4	NO	YES	NO
2106563	Tracheostomy, planned (separate procedure); younger than 2 years	Procedure	CPT4	NO	YES	NO
2106562	Tracheostomy, planned (separate procedure)	Procedure	CPT4	NO	YES	NO

List of negative control outcomes and OMOP Common Data Model ID

Supplementary Table 3: List of negative control outcomes and OMOP Common Data Model IDs.

Outcome ID	Outcome Name
436409	Abnormal pupil
199192	Abrasion and/or friction burn of trunk without infection
4092879	Absent kidney
436634	Acariasis
44783954	Acid reflux
75911	Acquired hallux valgus
137951	Acquired keratoderma
77965	Acquired trigger finger
376707	Acute conjunctivitis
4103640	Amputated foot
432595	Amyloidosis
73241	Anal and rectal polyp
437082	Ankylosing spondylitis
77650	Aseptic necrosis of bone
378424	Astigmatism
374923	Bell's palsy
4110709	Benign epithelial neoplasm of skin
133655	Burn of forearm
73560	Calcaneal spur
434327	Cannabis abuse
4213540	Cervical somatic dysfunction
381581	Chalazion
140842	Changes in skin texture
438531	Chondromalacia
81378	Chondromalacia of patella

432303	Cocaine abuse
4201390	Colostomy present
46269889	Complication due to Crohn's disease
134438	Contact dermatitis
78619	Contusion of knee
201606	Crohn's disease
260134	Croup
76786	Derangement of knee
4115402	Difficulty sleeping
443767	Disorder of eye due to diabetes mellitus
45757370	Disproportion of reconstructed breast
433111	Effects of hunger
441589	Endocarditis
200775	Endometrial hyperplasia
433527	Endometriosis
73008	Enthesopathy
4249170	Epicondylitis
4170770	Epidermoid cyst
4345472	Epstein-Barr virus disease
4092896	Feces contents abnormal
259995	Foreign body in orifice
196456	Gallstone
40481632	Ganglion cyst
4166231	Genetic predisposition
74855	Genital herpes simplex
433577	Hammer toe
441818	Hemangioma
4231770	Hereditary thrombophilia
440329	Herpes zoster without complication
4012570	High risk sexual behavior
4038835	Hodgkin's disease

4012934	Homocystinuria
441788	Human papilloma virus infection
380688	Hypoglycemic coma
4254542	Hypopituitarism
4201717	Ileostomy present
374375	Impacted cerumen
140480	Impetigo
4344500	Impingement syndrome of shoulder region
139099	Ingrowing nail
444132	Injury of knee
434926	Iridocyclitis
196168	Irregular periods
75576	Irritable bowel syndrome
432593	Kwashiorkor
434203	Late effect of contusion
438329	Late effect of motor vehicle accident
443285	Lesion of cervix
195873	Leukorrhea
440638	Lyme disease
4083487	Macular drusen
4156115	Malignant neoplasm of endocrine gland
4103703	Melena
4134455	Mononeuropathy
4209423	Nicotine dependence
377572	Noise effects on inner ear
136368	Non-toxic multinodular goiter
40480893	Nonspecific tuberculin test reaction
4215978	Onychomycosis
140648	Onychomycosis due to dermatophyte
438130	Opioid abuse
192606	Paraplegia

4091513	Passing flatus
4112731	Polyp of intestine
4202045	Postviral fatigue syndrome
373478	Presbyopia
46286594	Problem related to lifestyle
439790	Psychalgia
81634	Ptotic breast
253954	Pulmonary tuberculosis
4087647	Rectal mass
380706	Regular astigmatism
438688	Sarcoidosis
4169984	Scar
141932	Senile hyperkeratosis
196236	Septic shock
254443	Sjögren's syndrome
36713918	Somatic dysfunction of lumbar region
443172	Splinter of face, without major open wound
81151	Sprain of ankle
72748	Strain of rotator cuff capsule
378427	Tear film insufficiency
134461	Tietze's disease
437264	Tobacco dependence syndrome
4234533	Tonsillitis
4030042	Toxic goiter
81893	Ulcerative colitis
194083	Vaginitis and vulvovaginitis
140641	Verruca vulgaris
380038	Viral conjunctivitis
4291005	Viral hepatitis
4248870	Visceroptosis
440193	Wristdrop

Covariates included in large-scale PS model

There are substantial variations in the range, resolution, and coding of clinical covariates captured in different healthcare databases. This makes it difficult to define a set of covariates to control for that is consistent across databases. OHDSI's Common Data Model (CDM) nonetheless allows us to define a consistent criteria broad enough to include most potential confounders, from which we can select a relevant subset in a data-driven manner using a sparse regression technique. The table below describes our selection criteria in more detail.

Supplementary Table 4: Within each database, a covariate is included in our PS model if its concept type falls within any of the following OHDSI CDM concept categories.

- Demographics (age in 5-year increments and month of index date)
- Condition Occurrence (condition occurrence in lookback window)
  - in 180 days prior to index date
  - in 90 days prior to index date
  - in 30 days prior to index date
- Condition Era (span of time when person assumed to have condition)
  - ever
  - overlapping with index date
- Drug Exposure (drug occurrence in lookback window)
  - in 180 days prior to index date
  - in 30 days prior to index date
- Drug Era (span of time when person assumed to have drug)
  - in 180 days prior to index date
  - in 30 days prior to index date
  - ever
  - overlapping with index date
- Procedure Occurrence
  - in 180 days prior to index date
  - in 30 days prior to index date
- Observation
  - in 180 days prior to index date
  - in 30 days prior to index date
  - observations count in 180 days prior to index date
- Measurement
  - in 180 days prior to index date
  - in 30 days prior to index date
  - high measurement in 90 days prior to index date
  - low measurement in 90 days prior to index date
  - measurements count in 180 days prior to index date
- Concepts Count in 180 days prior to index date
- Risk Scores (Charlson, DCSI, CHADS2)
